# Supplementary material for: A wall-time minimizing parallelization strategy for approximate Bayesian computation
Source: PLoS One. 2024 Feb 22;19(2):e0294015. doi: 10.1371/journal.pone.0294015 (PMC10883530; doi:10.1371/journal.pone.0294015)
Supplement: S1 File — (PDF) [file pone.0294015.s001.pdf]

Supplementary information for:
A Wall-time Minimizing Parallelization Strategy for Approximate Bayesian Computation

Emad Alamoudi[1,*], Felipe Reck[1,*], Nils Bundgaard[2], Frederik Graw[2,3,4], Lutz Brusch[5], Jan Hasenauer[1,6,7], and Yannik Schälte[1,6,7]

[1] University of Bonn, Life and Medical Sciences Institute, 53113 Bonn, Germany
[2] Heidelberg University, BioQuant - Center for Quantitative Biology, 69120 Heidelberg, Germany
[3] Heidelberg University, Interdisciplinary Center for Scientific Computing, 69120 Heidelberg, Germany
[4] Friedrich-Alexander-University Erlangen-Nürnberg, Department of Medicine 5, 91054 Erlangen, Germany
[5] Technische Universität Dresden, Center of Information Services and High Performance Computing (ZIH), 01062 Dresden, Germany
[6] Helmholtz Zentrum München, Institute of Computational Biology, 85764 Neuherberg, Germany
[7] Technische Universität München, Center for Mathematics, 85748 Garching, Germany
[*] These authors contributed equally to this work.

Contents

1 Multiple proposal importance sampling **2**

1.1 Standard importance sampling 2

1.2 Effective sample size 3

1.3 Importance sampling with multiple proposal distributions 4

1.4 Subpopulation weighting 5

2 Implementation **6**

2.1 Algorithm implementation in pyABC 6

2.2 Technical specifications 6

3 Test models 6

3.1 (T1) Unbalanced Modes . . . 7

3.2 (T2) ODE Model . . . 7

3.3 (M1) Tumor Growth Model . . . 12

3.4 (M2) Liver regeneration model . . . 18

3.4.1 Simulation time variability . . . 26

1 Multiple proposal importance sampling

Let $\theta \in \mathbb{R}^{n_\theta}$ denote the latent parameters, $\pi(\theta)$ the prior, and $\pi(y|\theta)$ the likelihood which we assume to be able to sample data $y \in \mathbb{R}^{n_y}$ from, while direct evaluation is not possible. Let $y_{\text{obs}} \in \mathbb{R}^{n_y}$ be the observed data. By Bayes' Theorem, we get the posterior distribution

$$\pi(\theta|y_{\text{obs}}) = \frac{\pi(y_{\text{obs}}|\theta)\pi(\theta)}{\pi(y_{\text{obs}})} \propto \pi(y_{\text{obs}}|\theta)\pi(\theta)$$

of parameters given observed data.

Let $\varepsilon > 0$ and $d : \mathbb{R}^{n_y} \times \mathbb{R}^{n_y} \to \mathbb{R}_{\geq 0}$ be a distance metric. ABC methods can be interpreted to generate samples from the joint distribution

$$(\theta, y) \sim \pi_{\text{ABC},\varepsilon}(\theta, y|y_{\text{obs}}) \propto I[d(y, y_{\text{obs}}) \leq \varepsilon]\pi(y|\theta)\pi(\theta), \tag{1}$$

such that the target marginal of interest,

$$\pi_{\text{ABC},\varepsilon}(\theta|y_{\text{obs}}) \propto \int I[d(y, y_{\text{obs}}) \leq \varepsilon]\pi(y|\theta)\, dy \cdot \pi(\theta)$$

can be obtained by simple projection to the first component. $\pi_{\text{ABC},\varepsilon}(\theta|y_{\text{obs}})$ is an approximation of the actual parameter posterior, with $\pi(\theta|y_{\text{obs}}) = \lim_{\varepsilon \searrow 0} \pi_{\text{ABC},\varepsilon}(\theta|y_{\text{obs}})$ under mild assumptions.

1.1 Standard importance sampling

We employ importance sampling (ABC-IS) embedded in a sequential Monte-Carlo scheme (ABC-SMC). For a proposal distribution $g(\theta) \gg \pi(\theta)$ and a target population size N, we use an ABC-IS scheme as shown in Algorithm 1. In ABC-SMC, Algorithm 1 is then iterated multiple times for successively refined thresholds ε and proposals g. Here we focus on a single such iteration.

This form of ABC-IS generates samples from the distribution

$$(\theta, y) \sim G(\theta, y) \propto I[d(y, y_{\text{obs}}) \leq \varepsilon]\pi(y|\theta)g(\theta). \tag{2}$$

Here, the $g(\theta)$ is because we sample $\theta \sim g(\theta)$, further we simulate data y from the likelihood and discard particles not satisfying the distance condition, such that

$$y|\theta \sim I[d(y, y_{\text{obs}}) \leq \varepsilon]\pi(y|\theta).$$

while *less than N acceptances* **do**
  sample parameter $\theta \sim g(\theta)$
  simulate data $y \sim \pi(y|\theta)$
  accept θ if $d(y, y_{\text{obs}}) \leq \varepsilon$
compute weights $w_i = \frac{\pi(\theta^i)}{g(\theta^i)}$, for accepted parameters $\{\theta^i\}_{i\leq N}$
output: weighted samples $\{(\theta_i, w_i)\}_{i\leq N}$

Algorithm 1: Importance ABC algorithm.

Therefore, the importance weights, Radon-Nikodym derivatives, of the proposal (2) against the target (1) are given by

$$v(\theta, y) = \frac{\pi_{\text{ABC},\varepsilon}(\theta, y|y_{\text{obs}})}{G(\theta, y)} = \frac{C \cdot I[d(y, y_{\text{obs}}) \leq \varepsilon]\pi(y|\theta)\pi(\theta)}{C_G \cdot I[d(y, y_{\text{obs}}) \leq \varepsilon]\pi(y|\theta)g(\theta)} = \frac{C}{C_G}\frac{\pi(\theta)}{g(\theta)} = \frac{C}{C_G}w(\theta)$$

with normalization constants C, C_G. If all normalizations were known exactly, we could calculate the unbiased importance estimator of a test function f over the ABC posterior as

$$\mathbb{E}_{\pi_{\text{ABC},\varepsilon}(\theta,y|y_{\text{obs}})}[f] = \mathbb{E}_{G(\theta,y)}[vf] \approx \frac{1}{N}\sum_i v_i f(\theta_i, y_i).$$

In general, we however do not know C and C_G and thus v only up to normalization, and therefore need to employ the self-normalizing estimate

$$\mathbb{E}_{\pi_{\text{ABC},\varepsilon}(\theta,y|y_{\text{obs}})}[f] = \frac{\mathbb{E}_{G(\theta,y)}[wf]}{\mathbb{E}_{G(\theta,y)}[w]} \approx \frac{\frac{1}{N}\sum_i w_i f(\theta_i, y_i)}{\frac{1}{N}\sum_i w_i} = \sum_i W_i f(\theta_i, y_i)$$

with self-normalized weights

$$W_i := \frac{w_i}{\sum_j w_j},$$

which uses another Monte-Carlo approximation for the normalization constant. It is only asymptotically unbiased, converging almost surely as $N \to \infty$.

Remark: Were we instead to accept all particles irrespective of the distance, as some implementations do, we would need to regard $I[(y, y_{\text{obs}}) \leq \varepsilon]$ as part of the weighting, as in that case $G(\theta, y) \propto \pi(y|\theta)g(\theta)$ s.t. $v(\theta, y) = C \cdot I[d(y, y_{\text{obs}}) \leq \varepsilon]\pi(\theta)/g(\theta)$. In general, this interchangeability of importance and rejection sampling occurs in various places in ABC algorithms. Further note that the here presented scenario based on a uniform acceptance kernel $I[d(\cdot, y_{\text{obs}}) \leq \varepsilon]$, which is the most widely used one, naturally generalizes to arbitrary acceptance kernels $K_\varepsilon(\cdot, y_{\text{obs}})$.

1.2 Effective sample size

Importance sampling allows to e.g. better explore high-density regions by tailored proposals. However, as the particles need to be weighted, some contribute more to estimates than others, which can be interpreted as having a lower *effective sample size (ESS)* than a sample of the same size

directly from the target distribution. A common definition of the ESS can be motivated as follows: Consider a linear combination

$$S_N = \frac{\sum_{i\leq N} w_i X_i}{\sum_{i\leq N} w_i}$$

of i.i.d. random variables X_i of variance $\sigma^2 > 0$, with weights $w_i \geq 0$. The unweighted mean $\frac{1}{N_e}\sum_{i\leq N_e} X_i$ of N_e variables has variance σ^2/N_e. Equating, we obtain

$$\frac{\sigma^2}{N_e} \overset{!}{=} \text{Var}(S_N) = \frac{\sum_{i\leq N} w_i^2}{(\sum_{i\leq N} w_i)^2}\sigma^2 \Rightarrow \text{ESS} := N_e = \frac{(\sum_{i\leq N} w_i)^2}{\sum_{i\leq N} w_i^2} \tag{3}$$

as a scale-invariant quantification of the ESS of the weighted sum.

1.3 Importance sampling with multiple proposal distributions

Now assume we have multiple proposal distributions $\{g_l(\theta)\}_{l\leq L}$ instead of just one, and have generated $N_l > 0$ accepted particles $\{(\theta_i^l, w_i^l)\}_{i\leq N_l}$ from each proposal, with $N = \sum_l N_l$, giving a total population $P = \{\{(\theta_i^l, w_i^l)\}_{i\leq N_l}\}_{l\leq L}$ of size N. This generalizes the scenario presented in the main manuscript, where we have two proposal distributions, a preliminary one and a final one.

Assume $N_l/N \to \alpha_l \in [0,1]$ for $N \to \infty$. Denote the normalized importance densities $v^l(\theta, y) = \pi_{\text{ABC},\varepsilon}(\theta, y|y_{\text{obs}})/G_l(\theta, y) = C_l w^l(\theta, y)$ with normalization constants $C_l > 0$.

The goal is to for a test function f define a robust Monte-Carlo estimate making use of all samples. One possible estimator could be obtained by just ignoring the fact that multiple proposals were used and just throwing all samples and weights together:

$$\begin{aligned}
\mathbb{E}_{\pi_{\text{ABC},\varepsilon}(\theta,y|y_{\text{obs}})}[f] \xleftarrow{N\to\infty} \frac{\frac{1}{N}\sum_l \sum_{i\leq N_l} w_i^l f(\theta_i^l, y_i^l)}{\frac{1}{N}\sum_l \sum_{i\leq N_l} w_i^l} &= \frac{\sum_l \frac{N_l}{N}\frac{1}{N_l}\sum_{i\leq N_l} w_i^l f(\theta_i^l, y_i^l)}{\sum_l \frac{N_l}{N}\frac{1}{N_l}\sum_{i\leq N_l} w_i^l} \\
\xrightarrow{N\to\infty} \frac{\sum_l \alpha_l \mathbb{E}_{G_l(\theta,y)}[w^l f]}{\sum_l \alpha_l \mathbb{E}_{G_l(\theta,y)}[w^l]} &= \frac{\sum_l \alpha_l C_l^{-1}\mathbb{E}_{\pi_{\text{ABC},\varepsilon}(\theta,y|y_{\text{obs}})}[f]}{\sum_l \alpha_l C_l^{-1}} \\
= \mathbb{E}_{\pi_{\text{ABC},\varepsilon}(\theta,y|y_{\text{obs}})}[f].
\end{aligned} \tag{4}$$

Equation (4) shows that we obtain an asymptotically unbiased estimate, however the subpopulations are weighted by the normalization constants C_l, which is in general not meaningful, unless these carry an appropriate interpretation. Further, this joint estimator does not account for the difference in importance estimator quality of the various G_l.

Instead of (4), we suggest to first consider each subpopulation separately, and obtain a joint estimate as a weighted sum of estimators

$$\begin{aligned}
\mathbb{E}_{\pi_{\text{ABC},\varepsilon}(\theta,y|y_{\text{obs}})}[f] = \sum_l \beta_l \mathbb{E}_{G_l(\theta,y)}[v^l f] &= \sum_l \beta_l \frac{\mathbb{E}_{G_l(\theta,y)}[w^l f]}{\mathbb{E}_{G_l(\theta,y)}[w^l]} \\
&\xleftarrow{N\to\infty} \sum_l \beta_l \sum_{i\leq N_l} W_i^l f(\theta_i^l, y_i^l)
\end{aligned} \tag{5}$$

with coefficients β_l s.t.

$$\sum_l \beta_l = 1, \tag{6}$$

and

$$W_i^l := \frac{w_i^l}{\sum_{j \le N_l} w_j^l}$$

subpopulation-wise self-normalized weights.

1.4 Subpopulation weighting

The β_l are free parameters and should be chosen to yield a robust estimator. A straightforward choice is

$$\beta_l = \alpha_l \approx \frac{N_l}{N}, \tag{7}$$

i.e. to normalize the contribution of each proposal by the number of samples generated from that proposal.

As the proposal quality can vary, this weighting may not be ideal. Instead of (7), we suggest to choose the β_l to e.g. maximize the overall ESS, in order to obtain a robust estimator. Here, (3) takes the shape

$$\mathrm{ESS} = \frac{(\sum_l \sum_{i:l} \beta_l W_i^l)^2}{\sum_l \sum_{i:l} (\beta_l W_i^l)^2} = \frac{1}{\sum_l \beta_l^2 \sum_{i \le N_l} (W_i^l)^2}. \tag{8}$$

Denote for short $Q_l := \sum_{i \le N_l} (W_i^l)^2$, the diagonal matrix $Q := \mathrm{diag}(Q_1, \ldots, Q_L) \in \mathbb{R}^{L \times L}$, and the column vectors $\beta := (\beta_1, \ldots, \beta_L)^T \in \mathbb{R}^L$ and $\mathbb{1} := (1, \ldots, 1)^T \in \mathbb{R}^L$. Without loss of generality, assume that $Q_l > 0$ for all l, otherwise just set $\beta_l = 0$. Then, maximizing (8) subject to (6) is equivalent to the quadratic form with linear equality constraint

$$\begin{aligned} &\text{minimize} \quad \beta^T Q \beta \\ &\text{subject to} \quad \mathbb{1}^T \beta = 1. \end{aligned} \tag{9}$$

Evaluating $\nabla_{\beta,\lambda}[\beta^T Q \beta + \lambda(\mathbb{1}^T \beta - 1)]$, using a Lagrange multiplier $\lambda \in \mathbb{R}$, readily shows that the unique solution of (9) is given by the positive-definite linear system

$$\begin{pmatrix} 2Q & \mathbb{1} \\ \mathbb{1}^T & 0 \end{pmatrix} \cdot \begin{pmatrix} \beta \\ \lambda \end{pmatrix} = \begin{pmatrix} 0 \\ 1 \end{pmatrix} \Leftrightarrow \begin{pmatrix} \beta \\ \lambda \end{pmatrix} = \begin{pmatrix} \frac{Q^{-1}\mathbb{1}}{\mathbb{1}^T Q^{-1} \mathbb{1}} \\ \frac{-2}{\mathbb{1}^T Q^{-1} \mathbb{1}}. \end{pmatrix}$$

Written out, we obtain

$$\beta_l = \frac{\frac{1}{\sum_{i \le N_l} (W_i^l)^2}}{\sum_k \frac{1}{\sum_{i \le N_k} (W_i^k)^2}} = \frac{\mathrm{ESS}(\{W_i^l\}_{i \le N_l})}{\sum_k \mathrm{ESS}(\{W_i^k\}_{i \le N_k})}, \quad l = 1, \ldots, L,$$

i.e. the overall ESS is maximized by setting the contribution of sub-population l proportional to its ESS, which intuitively makes sense.

2 Implementation

2.1 Algorithm implementation in pyABC

The ABC-SMC algorithm and all samplers used and developed in this work have been implemented and made available as part of the python-based open-source package pyABC (`https://github.com/icb-dcm/pyabc`). The sampler implementation uses the *Redis* package (`https://redis.io`) as broker between main process and workers on a distributed high-performance computing (HPC) architecture.

Before the analysis, a Redis server is set up. To this server the desired number of workers is then connected. Workers can also be added interactively during the analysis. In addition, there is a process for the main analysis, the ABC-SMC algorithm, which submits in each generation simulation tasks to the server, which broadcasts them to the workers and collects results, to be read in again by the main process. The whole process of environment setup and analysis has been fully automated for HPC infrastructure.

Simulation tasks are broadcast by the server via dedicated port channels, which workers listen on if they are idle. Workers return simulation results via a queue, which is continually collected from by the main process. Start times are tracked via shared variables to ensure the main process is aware which tasks need to be waited for.

2.2 Technical specifications

For our tests we used Anaconda 3, Python 3.8, with package versions pyABC 0.12.07, tumor2d 1.0.0, Morpheus 2.2.0 [1]. Analyses of model T1 was performed on the Juelich Supercomputing Center (JSC), Juwels cluster, standard compute nodes, specification of which are 2 $\times$ Intel Xeon Platinum 8168 CPU, 2 $\times$ 24 cores, 2.7 GHz 96 (12 $\times$ 8) GB DDR4, 2666 MHz.

Analyses of model T2, M1, and M2 were performed on the Bonna cluster at the University of Bonn. A standard node has the specification of 2 $\times$ 16 cores, 6 GB RAM per core, and 2x480GB SSD. For the different models, we used 1-9 nodes for T1 and 1-8 nodes for T2, M1 and M3.

3 Test models

To test the correctness and the performance of the new concept, we used 4 models as described in the Main Manuscript, Table 1, which we describe in further detail in this section. First, we used LA on some basic toy models (T1 and T2) to examine the general behavior and some specific properties. Later, we also performed tests on more complex application examples (M1 and M2) to test the performance in realistic settings.

Table 1: List of fitted parameters of model T1. All parameters have been transformed to a logarithmic scale with base 10.

Parameter	Description	Prior
$\log_{10}(\theta)$	A squared parameter	U(-2,-4)

3.1 (T1) Unbalanced Modes

Usually, the run-time of a single simulation will not be independent of the parameter candidate but instead, be somehow correlated to the region of the parameter space. High variance of the run-time paired with correlation between the simulation time and the candidate could possibly lead to a strong imbalance in the preliminary proposal. This raises the question of what happens when it is likely that the preliminary population is strongly biased.

To analyze this phenomenon, we constructed a two-mode scenario by considering a model that simply squares its only parameter, i.e. $y = \theta^2$ and then adds additive noise to that. Assuming the observed data consist of one observation with value 1 and that the prior is uniformly distributed on $[-2, 4]$, we get two parameters that could equally be true, -1 and $+1$, see Table 1. We added a shorter idle time sampled from a log-normal distribution with mean 0.05 and σ^2=0.025 to the negative mode and a larger one (mean=1 and σ^2=0.5) to the positive mode. Given a large number of parallel workers and a comparably small population sizes, it is thus possible that the preliminary population $\hat{P}_{t-1}$ consists entirely of samples from the negative side. Depending on the shape of the thus created preliminary proposal $\tilde{g}_t$, it is then possible that it only proposes samples from the negative side. It can happen that enough of these get accepted, such that the subsequent accepted population P_t consists only of samples from the negative side.

3.2 (T2) ODE Model

While ODE based models are not the main application area of ABC, it is fairly simple to create an example for which we can easily verify the correct result. Parameter inference for such a model is also not as time intensive as real life application examples. Therefore, we can perform it several hundred times with the new concept and compare the results to the ones of established approaches, making this a good test model to perform sanity checks on the new algorithm.

Our basic ODE-model with two parameters describes the inter-conversion between two species x_1, x_2, with rates θ_1, θ_2. It is given by

$$\frac{d}{dt}x_1 = -\theta_1 x_1 + \theta_2 x_2 \tag{10}$$

$$\frac{d}{dt}x_2 = \theta_1 x_1 - \theta_2 x_2. \tag{11}$$

Using this ODE, we can immediately verify that the results converge towards the same value as the original version for which the asymptotical correctness is known. In order to not have a fully

deterministic system and to consider that in reality measurements are not perfectly accurate, we added multiplicative normal noise $\sim \mathbb{N}(1, 0.03)$ to each model evaluation.

To analyse the run-times and the effect of heterogeneous simulation times, we used the same ODE and added some idle time to each model call. This idle time was chosen in a way that imitates some single simulations taking vastly longer than the average value. As distribution for t_{idle} we employed a log-normal one $t_{idle} \sim \text{lognormal}(\mu_n, \sigma_n^2)$, where μ_n, σ_n^2 are the mean and variance of the underlying normal distribution. μ_n, σ_n^2 were chosen such that the real variance σ^2 takes values between 0.25 and 4 and the real mean μ is constantly equal to 1 for the different values of σ^2, i.e.

$$\sigma_n^2 = 2\log\left(\frac{\sqrt{1^2+\sigma^2}}{1}\right), \quad \mu_n = \log(1) - \frac{\sigma_n^2}{2}. \tag{12}$$

We used as prior a uniform distribution on the unit square $\pi \sim U([0,1]^2)$, initial values $(x_1, x_2)(0) = (1, 0)$, and as true parameters $\theta_1 = e^{-2.5} \approx 0.0821$, $\theta_2 = e^{-2} \approx 0.135$, so here a point close to one corner of the prior distribution see Table 2. As observed data y_{obs} we considered the trajectory of species x_2 at time points $0, 1, \ldots, 10$. As distance function we used an l_1 norm. Candidates are accepted if the distance between their simulated data and the observation falls below a fixed ε-threshold for each generation, with pre-defined thresholds of $\varepsilon_1, ..., \varepsilon_8 = 8, 4, 2, 1, 0.7, 0.5, 0.33, 0.25$.

Table 2: List of fitted parameters of model T2. All parameters have been transformed to a logarithmic scale with base 10.

Parameter	Description	Prior
$\log_{10}(\theta_1)$	Conversion rate of x_1	U(0,1)^2
$\log_{10}(\theta_2)$	Conversion rate of x_2	U(0,1)^2

Results – Correctness

Using (T2) we generated 13 replicas of different configurations of both using DYN and LA scheduling for each population size on 32 to 256 workers.

Both scheduling approaches seem to return equal results (see Figure 4) when taking into account that any two runs will always slightly differ due to the innate random nature of the sampling, the noise and the run-time.

(A) demonstrates how the mean for both DYN and LA converges to the same value as we increase the population size. At the same time, (B) shows that also the standard deviation within individual distributions decreases, meaning that, as N increases, both posteriors peak more sharply at the same value. (C) makes it clear that a stronger variances for t_{idle} does not affect the mean, even though there is a larger fraction of preliminary particles expected. In (D), one can see that LA does not seem to substantially affect the effective sample size either even though we here used $N = 20$, which, on 256 workers, makes it likely that several generations are sampled completely from the preliminary. In E and F, we can see the fraction $\tilde{N}/N$ of accepted samples in the final population at

$t = n_t$ that were generated using the preliminary proposal $\tilde{g}_{n_t}(\theta)$. In G, we can see a comparison of the posterior distribution of the different scheduling. One can appreciate the nice agreement in the shape of the posterior from different scheduling strategies. By showing that the preliminary and the final proposal both considerably contribute to the posterior distribution, we get a strong indication that everything works as intended. And indeed, in many of the relevant scenarios the amount of particles that were based on the preliminary in the last generation ends up making up roughly half of the population, which is returned as output by the ABC-SMC algorithm (see Figure S1).

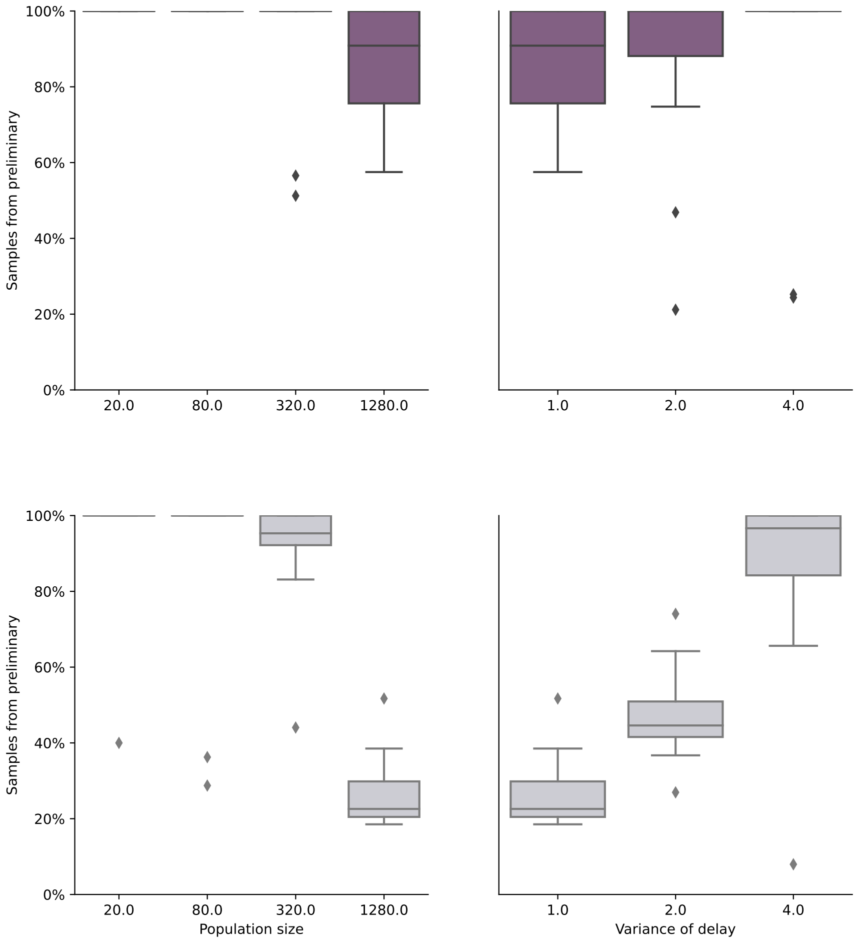

Figure S1: Fraction of particles sampled from the preliminary proposal in the last generation (for $\sigma^2 = 1$ (left) and $N = 1280$ (right)) for LA Prel (upper row) and LA Past (second row).

.

In Figure S1, one can also observe how the fraction of particles sampled from the preliminary decreases as the population size increases. This makes sense, since keeping the number of workers constant results in a more or less constant amount of resources used for the preliminary sampling between the N-th acceptance in a generation and the last worker to finish working on that generation. So, there should be a roughly constant number of preliminary acceptances opposite to an

increasing size of the total population.

Similarly, a higher run-time variance increases the fraction of preliminary particles, as this results in more time between the N-th acceptance and the last simulation of a generation.

Results – Run-Time

To analyze how much effect the new scheduling can have on the wall time, we ran the same model on different population size to worker ratios. First, we used an idle time with variance $\sigma^2 = 1$, the same population sizes as above, i.e. between 20 and 1280 and ran each of those on 1, 2, 4, and 8 nodes with 32 workers each, so on 32 to 256 workers. For a run-time variance of $\sigma^2 = 1$, we started with 13 repetitions of each scheduling approach (Except for STAT, where we used only 3 repetitions for time-preserving purposes) for each population size on 256 workers (see Figure 5). For $\sigma^2 = 2$, run-times were generally higher (see Figure S2).

We can observe a substantial speed-up when using LA instead of DYN scheduling (see Figure 5 and S2), whenever the population size is about as large or slightly larger than the amount of workers. In the best case, the LA approach decreased the wall time by nearly a factor of two when compared to the dynamic scheduling runs. On the other hand, the difference in efficiency is much less apparent, or even almost trivial, in case the two factors, population and worker size, are vastly different.

In the case where the amount of workers is substantially larger than our population size, only few simulations remain for each worker. This can go as far as even having enough preliminary acceptances to complete the next generation even before the previous one is finished leaving all further simulations retrospectively useless, which results in LA scheduling also having a poor parallel efficiency. Nonetheless, in that case there is still a substantial acceleration when compared to established approaches as LA can almost complete two generations in the time it takes to complete one using DYN.

When the population size is multiple times larger than the amount of available workers, dynamic scheduling already performed very well, leaving little room for improvement. However, even in that scenario we do not have any changes to the negative, so any additional computation necessary to enable the sampling from the preliminary population appears always worth the effort.

Further, using the same setup, we examined the effect of the run-time variance of the single evaluations on the speed-up. For that we ran the same tests as before with an idle time variance of $\sigma^2 = 2$; and indeed results (see Figure S2) seem to indicate that the achieved acceleration is even slightly higher than for the less varying run-time with $\sigma^2 = 1$.

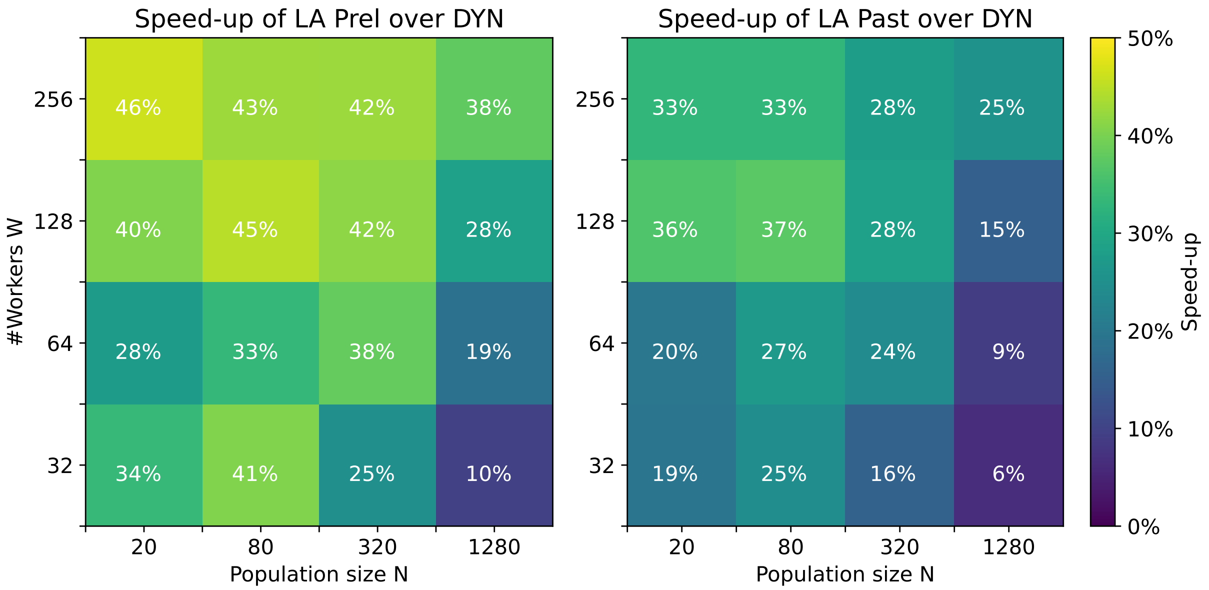

Figure S2: The speed-up for LA Prel (left) and LA Past (right) with $\sigma^2 = 2$

Finally, both DYN and LA scheduling scale better than STAT when using more computational resources (See Figure S3)

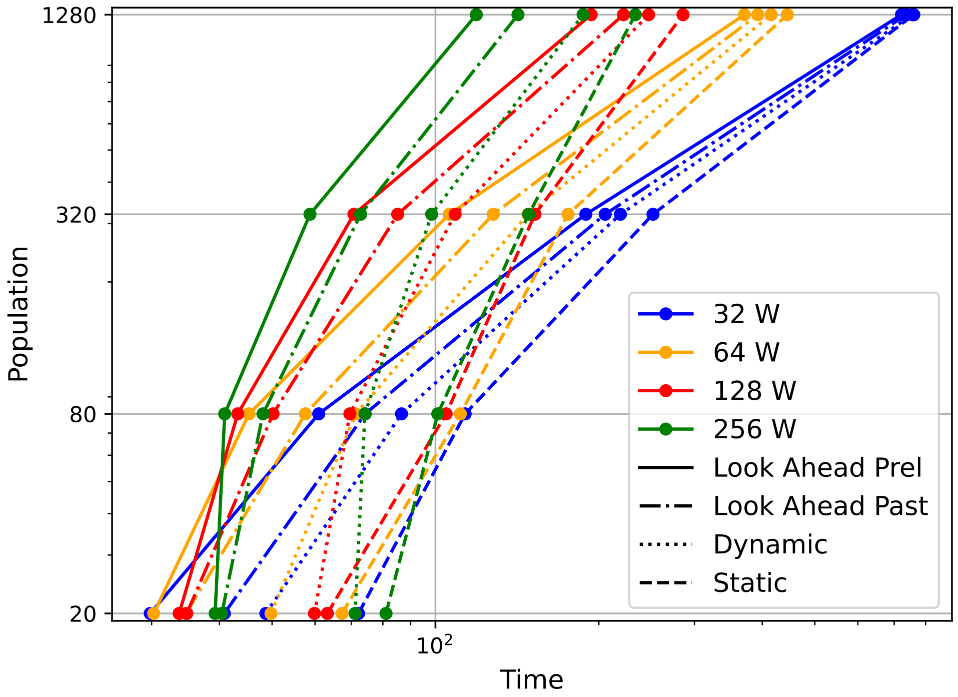

Figure S3: The time taken by different scheduling methods on different population sizes and numbers of workers.

3.3 (M1) Tumor Growth Model

The tumor growth model (M1) is our first test instance based on a real life example. The model was developed mainly in [2] and aims to describe the growth of a tumor spheroid while taking into account its spacial structure. For example, the proliferating cells are almost exclusively the ones in the outer rim of the spheroid whereas the ones contained in the core are mostly necrotic.
As hybrid discrete-continuous model it uses different mathematical tools trying to accurately depict a real life biological system.

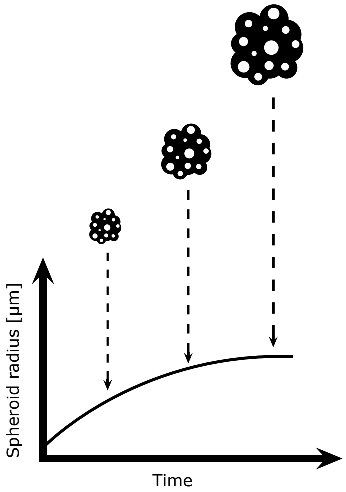

An agent-based approach with stochastic interactions between separate particles in a system, is used to model the relations between the individual cells, while a system of PDEs describes the extracellular matrix. At the same time, mechanisms like cell division and cell death are modeled using a continuous time Markov process. For further details about the model see [2, 3] and for some biological background see e.g. [4, 5].

The version we used as test instance is a two dimensional implementation of the tumor growth model with seven parameters see Table 3. It is available in the python package *tumor2d* (`https://github.com/ICB-DCM/tumor2d`). Even using several hundred workers and a population size of e.g. 1000, the parameter inference for the tumor growth model takes hours to days, making it close to impossible to run the simulation on anything but large-scale parallelized infrastructure and strictly necessary to employ an efficient workload distribution scheme. Yet, the run-time is not yet as exorbitantly high as for some other models, such that it was possible to perform several runs to obtain at least some reliability in the results. This was particularly important as the trajectories of the tumor growth model underlie stochastic fluctuations, especially in the early phases, as there the number of cells is still very limited. Towards the end, the cell numbers are far higher and the fluctuations are much less severe due to the averaging effect of the Law of Large Numbers.

Results – Correctness

We ran model M1 with population sizes ranging from 250 to 1000, employing two different amounts of workers 128 and 256. The acceptance criteria for candidates of a generation are adaptively chosen after the previous one finishes as the 50% quantile of the previous accepted distances. The run finishes after a generation t in which the threshold ε_t falls below a certain value, in our runs $\varepsilon_{n_t} \leq 700$. Every time one scenario was run, one instance with dynamic scheduling, one with LA Prel, and one with LA Past scheduling was performed with the same setup to directly compare the results.

Table 3: List of fitted parameters of model M1. All parameters have been transformed to a logarithmic scale with base 10.

Parameter	Description	Prior
$\log_{10}(k^{\text{div}}_{\text{max}})$	Division rate	U(-3,-1)
$\log_{10}(L_{\text{div}})$	Division depth	U(-5,0)
$\log_{10}(L_{\text{init}})$	Initial spheroid radius	U(1,3)
$\log_{10}(q_{\text{init}})$	Initial quiescent cell fraction	U(0,1.2)
$\log_{10}(k^{e}_{\text{pro}})$	ECM production rate	U(-5,0))
$\log_{10}(k^{e}_{\text{deg}})$	ECM degradation rate	U(-5,0))
$\log_{10}(e_{\text{div}})$	ECM division threshold	U(-5,0))

During the runs, no substantial deviations in the quality of results were observed (see Figure S4 and Figure S5; detailed results of the other runs can be found in the supplementary code).

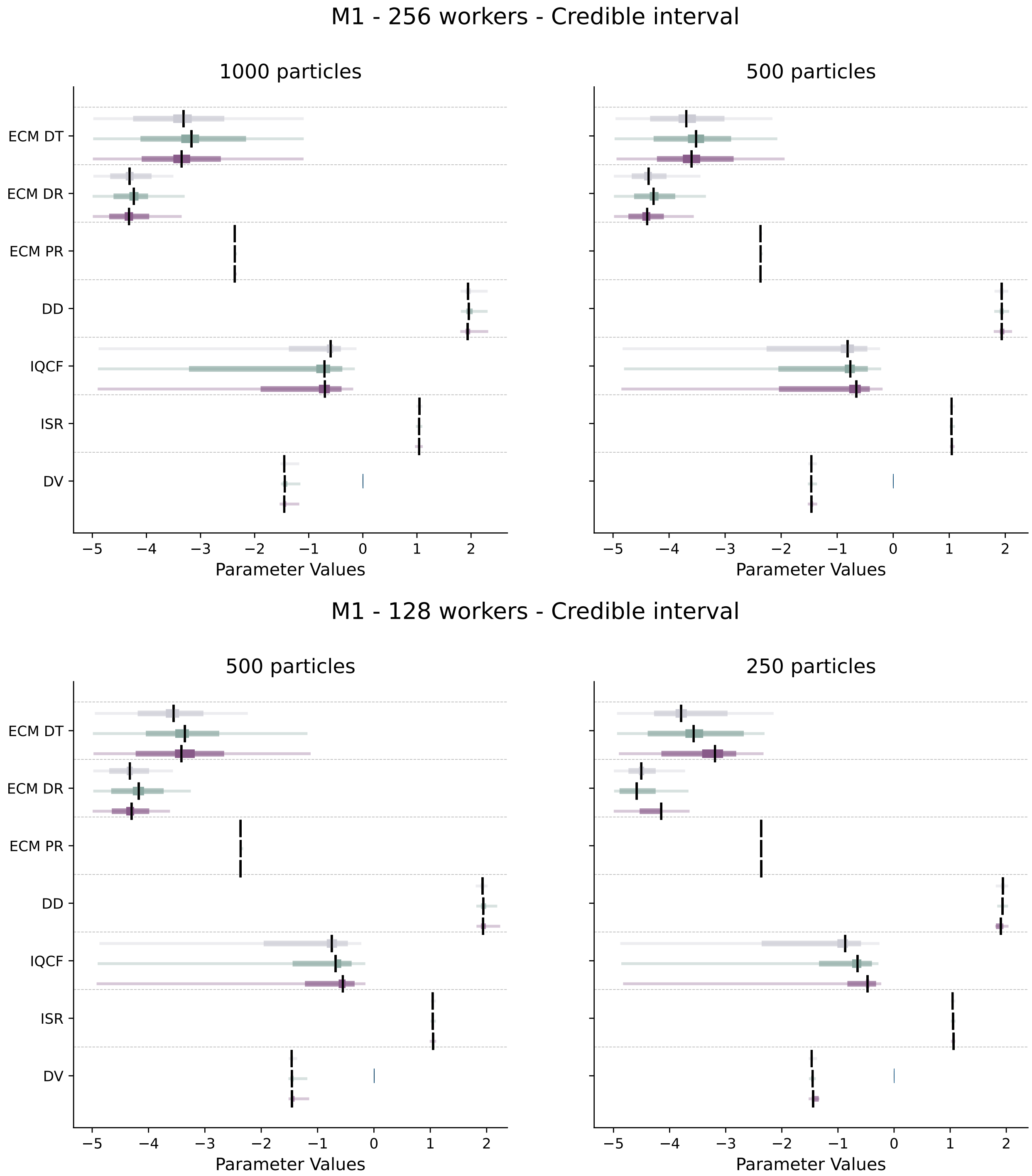

Figure S4: The credible interval of the model M1 with population size 1000, 500, 250 on 128 and 256 workers.

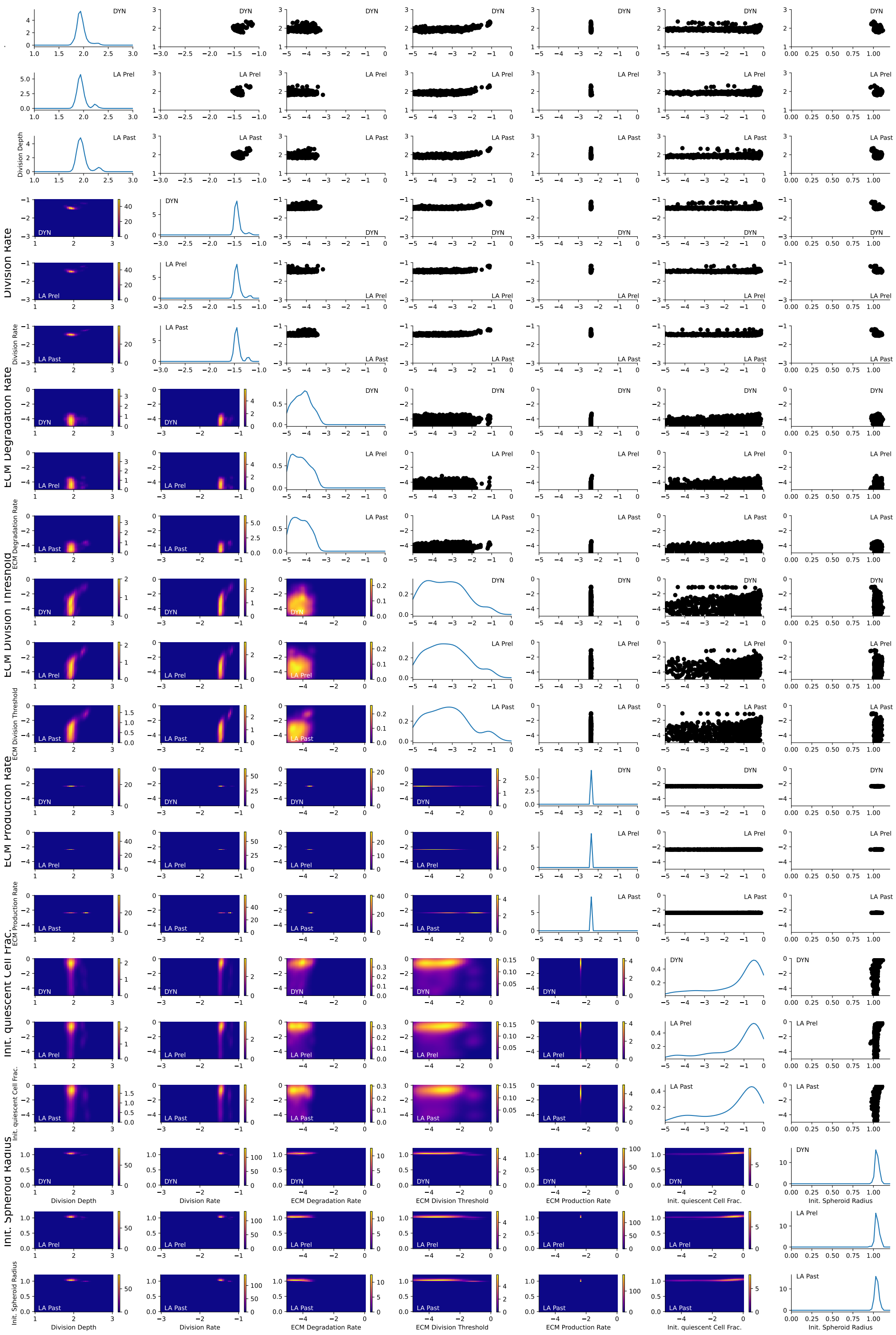

Figure S5: Direct comparison of the posterior distributions for all seven parameters of (M1) for a run with $N = 1000$ on $W = 256$ workers using DYN, LA Prel, and LA Past scheduling. The parameters with sharp peaks are visibly at the same location and if the inference returned a broader distribution, it did so in all cases.

While the fraction of preliminary particles tends to decrease over the course of the inference, it varies strongly from generation to generation (see Figure S6). As the time between the N-th acceptance in generation t and the last worker to finish is expected to be more or less constant, we have a similar amount of evaluations from the preliminary proposal in each generation. Generally, the acceptance rate decreases with the epsilon threshold however, and this also holds for the preliminary proposal based candidates. So, as more total evaluations are necessary to reach the desired number of accepted particles N, a smaller fraction of those should end up being sampled from the preliminary as the generations progress and the acceptance rate decreases.

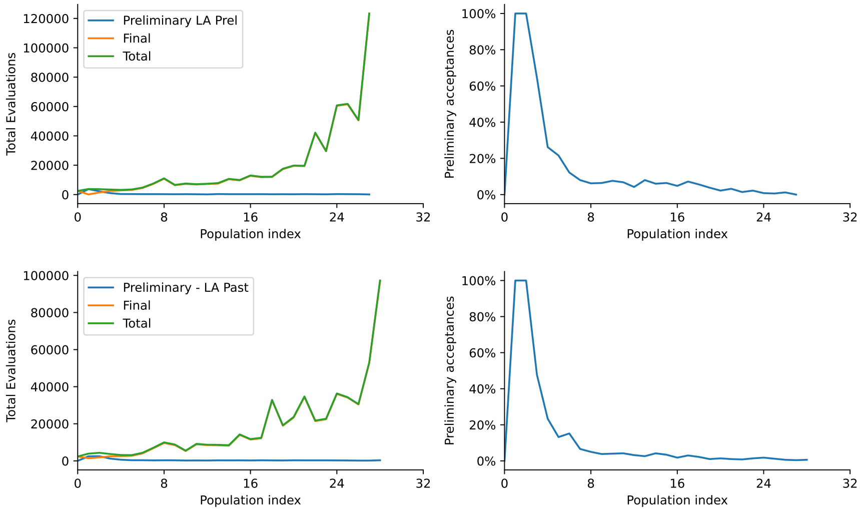

Figure S6: Total evaluations and fraction of accepted particles of M1 based on the preliminary population in each generation in the LA Prel (top) and LA Past (bottom) runs of the same run as in Figure S5

Results – Run-Time

For more complex models, it is usually far more effective to use the adaptive epsilon schedule mentioned in the previous section. That however, leads to a different final acceptance threshold in every run, which strongly affects the run-time. Together with the very stochastic start of the tumor growth model, this yields a high variance of the wall times, making a direct comparison more difficult. Nonetheless, we can for example observe the time point after which each epsilon value is achieved.

In the more extreme cases, it also occurred that the LA scheduling took slightly longer than the corresponding DYN run, but similarly also that the LA run decreased the wall time by a factor of more than 2 when compared to the DYN scheduling based one (see Figure S7).

These stronger differences in wall time usually seem to be traceable to one single generation taking vast amounts of time. Those long generations occur in all scheduling variants and exist most likely because the epsilon for that generation was chosen too optimistically.

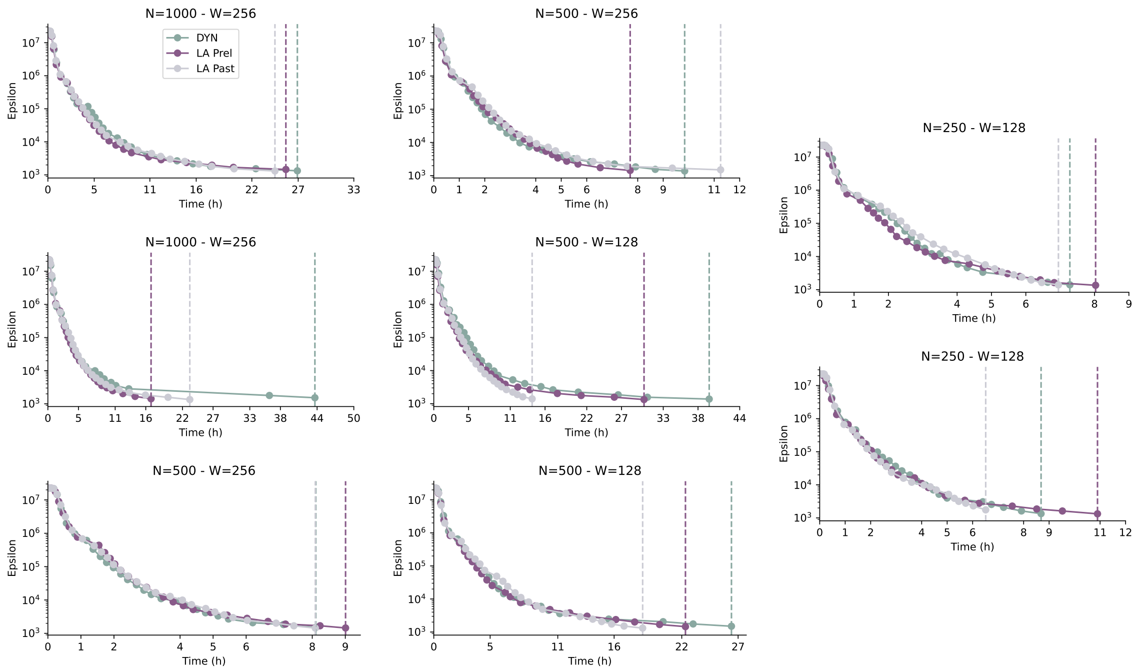

Figure S7: Development of the acceptance threshold over time for the different runs for model M1. These are all the 8 runs we performed.

On average, it seems that acceleration varies based on the population size and the number of workers to be used. Over the 8 times we have executed the inference of the tumor model with an adaptive epsilon schedule, we observed a mean acceleration of 21%, with the median value being 23% for LA Past.

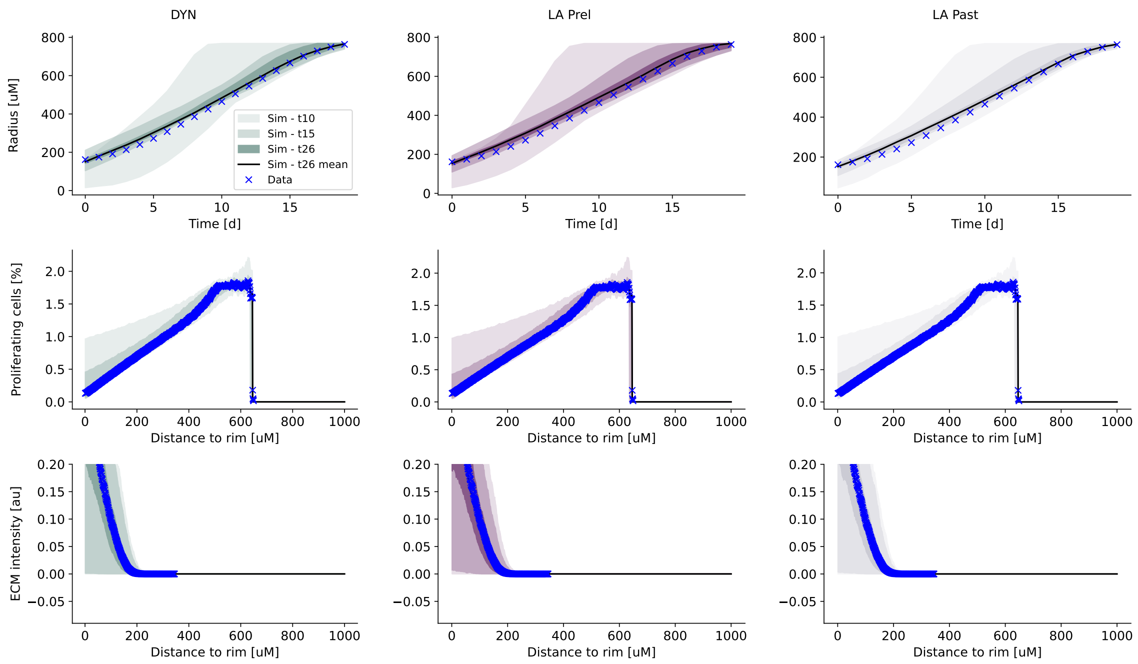

Figure S8: Comparison of best parameter fit for M1 using DYN (left), LA Prel (middle), and LA Past (right) for a population size of N= 1000

3.4 (M2) Liver regeneration model

M2 is a model of YAP regulation by mechanical stimulation through expansion of the bile canaliculi (BC) [6]. A single realisation of a model varies greatly based on parameter values ranging from 3 to 3,000 seconds. This model has 14 unknown parameters (see Table 4)and two observables, namely nuclear YAP and total YAP intensities which were quantified from image tiles covering an entire portal and central vein. The yes-associated protein (YAP) is an activator that activates the Hippo pathway that plays an important role in liver regeneration.

Two sub-models were used to describe the changes of osmotic pressure and the concomitant activation of YAP after PH. The first sub-model is a biophysics-based model to predict the local mechanical stress and apical membrane strain that result from the alteration of osmolyte (bile acid) load in the BC network after partial hepatectomy. It considers the spatial geometry of the BC within the portal and central vein axis of the lobule. Sub-model 2 is a biochemistry-based model that predicts the cellular response of YAP to the local mechanical stress.

Results – Correctness

Several fitting configurations were done to model (M2) with population sizes ranging from 250 to 1000, with two different amounts of workers, 128 and 256. The acceptance criteria for particles were adaptively set to be the 30% quantile of the previously accepted distances. The run set to be finished after the discrepancy threshold ε_t falls under some values. The value was set differently

Table 4: List of fitted parameters of model M2. All parameters have been transformed to a logarithmic scale with base 10.

Parameter	Description	Prior
$\log_{10}$(k1)	Max. flux of SENSOR activation	U(1,3)
$\log_{10}$(k2)	Max. flux of SENSOR inactivation	U(0,2)
$\log_{10}$(k3)	Factor of YAP synthesis rate	U(-0.76,1.23)
$\log_{10}$(k4)	YAP inactivation rate	U(-1.95,0.04)
$\log_{10}$(k5)	YAP activation rate	U(1,4)
$\log_{10}$(k6)	Inact. YAP binding rate to SF	U(-1.74,0.25)
$\log_{10}$(k7)	Inact. YAP unbin. rate from SF	U(1,4)
$\log_{10}$(k8)	YAP export rate from nucleus	U(1,4)
$\log_{10}$(k9)	YAP import rate into nucleus	U(-1.76,0.23)
$\log_{10}$(k10)	YAP degradation rate	U(-0.95,1.04)
$\log_{10}$(k11)	Inact. YAP degradation rate	U(0.47,2.47)
$\log_{10}$(k12)	M-M const. of SENSOR activation	U(-4,-2)
$\log_{10}$(k13)	M-M const. of SENSOR inactivation	U(-1.6,0.40)
$\log_{10}$(k14)	Intensity normalization total	U(-1,1)

based on different configurations ranging from 1.4e+03 to 2.0e+03. Similar to model (M1), the three different samplers, DYN, LA Prel, and LA Past, were executed on similar configurations to ensure a fair comparison.

Assessing the quality of the result, there were no substantial differences of the results during the run (see Figure S9 and S10 and Figure S11; detailed results of the other runs can be found in the supplementary code).

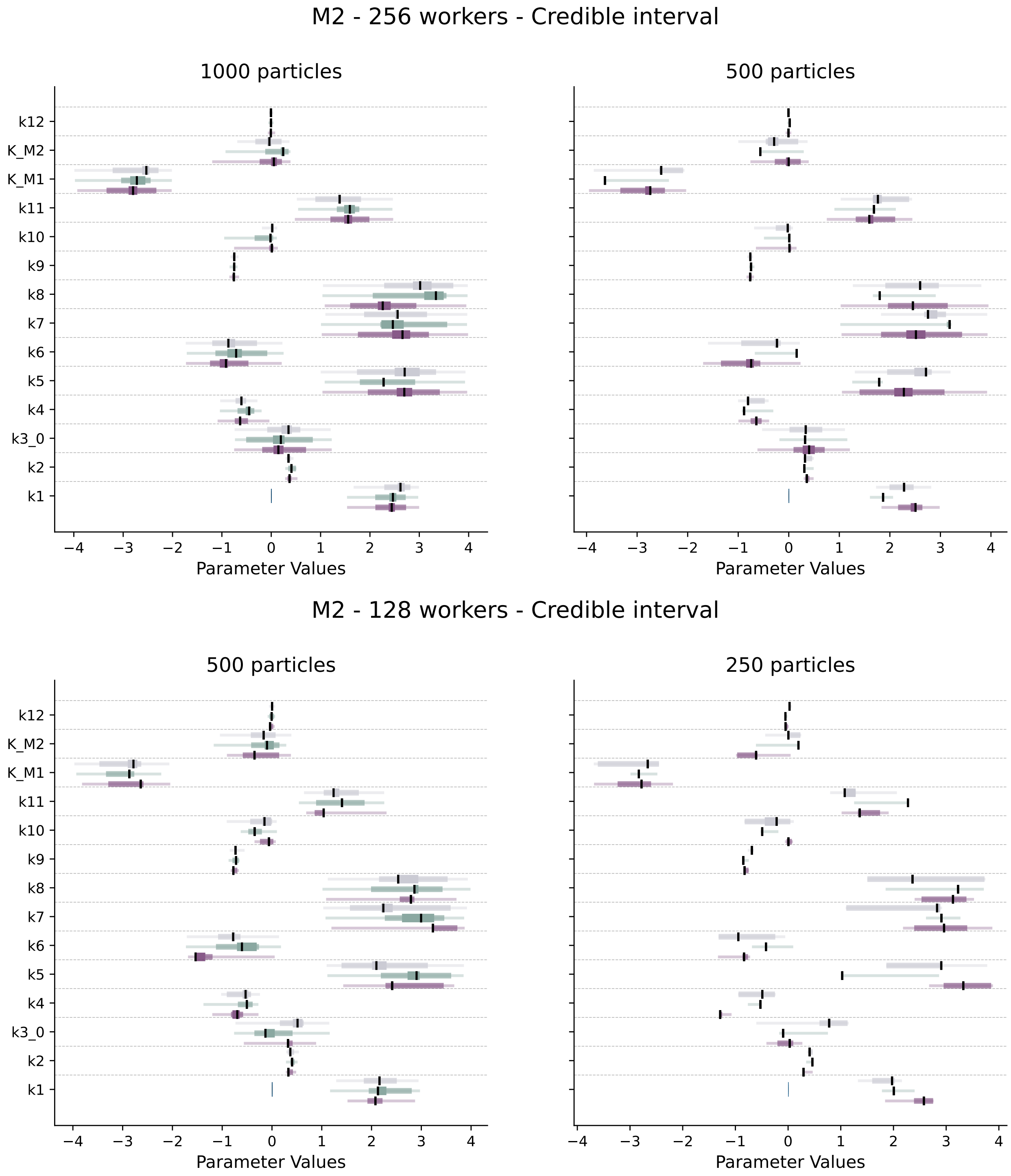

Figure S9: The credible interval of the model (M2) with population size 1000, 500, 250 on 128 and 256 workers. A similar maximum simulation time was used here as in Figure S14 (900 seconds as maximum simulation time)

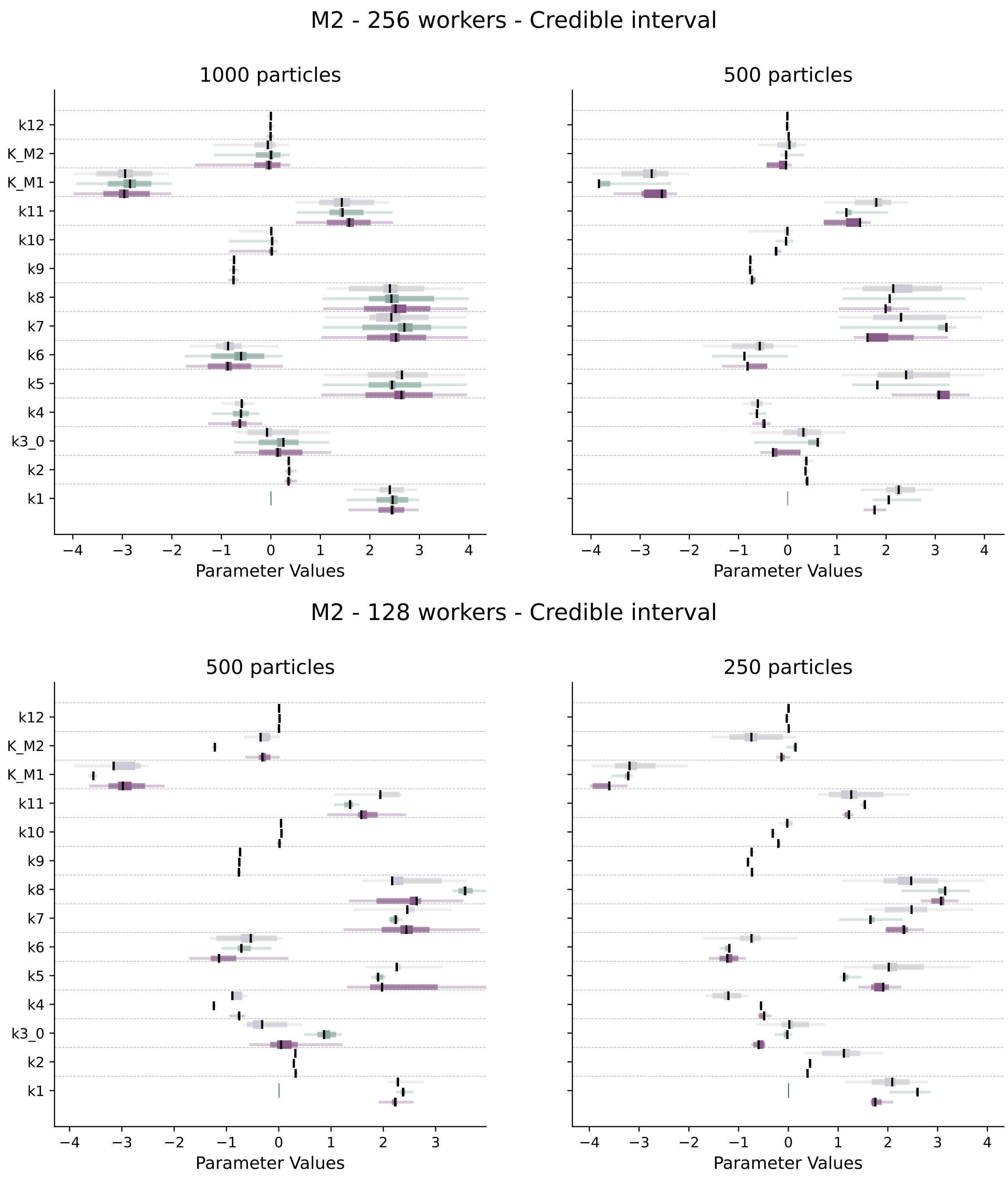

Figure S10: The credible interval of the model (M2) with population size 1000, 500, 250 on 128 and 256 workers. A maximum simulation time was used here as in Figure 6 (1800 seconds as maximum simulation time).

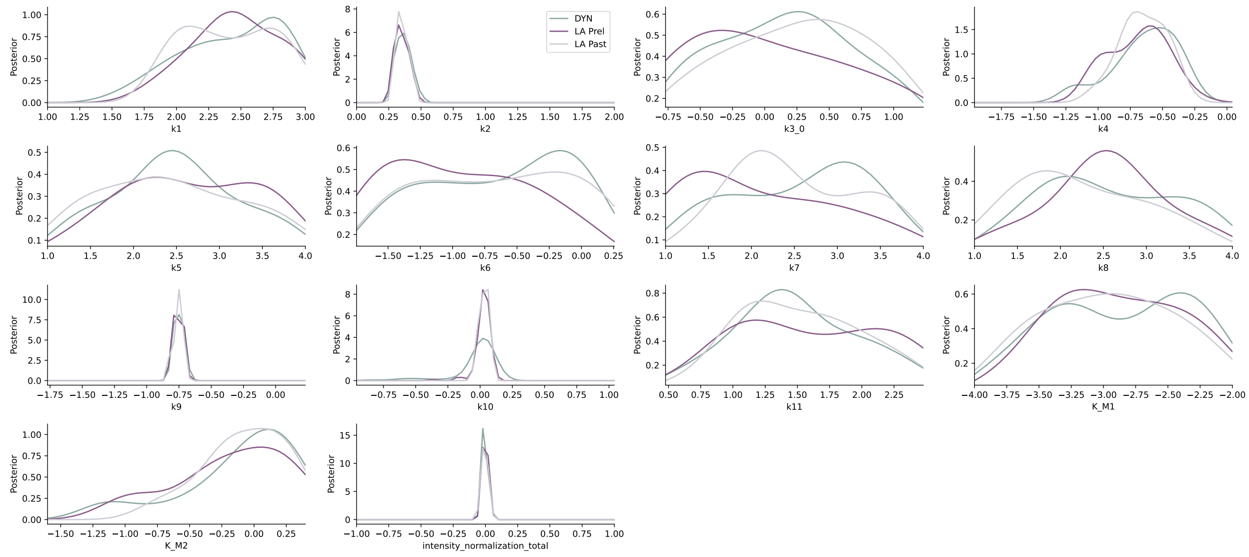

Figure S11: Direct comparison of the posterior distributions for all 14th parameters of (M2) for a run with $N = 1000$ on $W = 256$ workers using DYN, LA Prel, and LA Past scheduling. The sharp peaks of the parameters are visibly at the same location, and if the inference yielded a more broader distribution, it does. It did so in all cases..

Similar to model (M1), the fraction of M2 model preliminary particles decrease over the course of the inference, While the fraction of preliminary particles tends to decrease over the course of the inference (see Figure S12).

The fluctuations of this fraction exist because the acceptance threshold, and thus the acceptance rate, decreases less consistently than when using a static epsilon schedule, as can at least partially be observed in Figure S15.

As a large fraction of the population is based on the preliminary proposal for a substantial part of the generations, it shows that these preliminaries have a large effect on the LA version of the ABC-SMC algorithm. Nonetheless, the posteriors are consistently similar, indicating that everything works as intended and no bias was introduced.

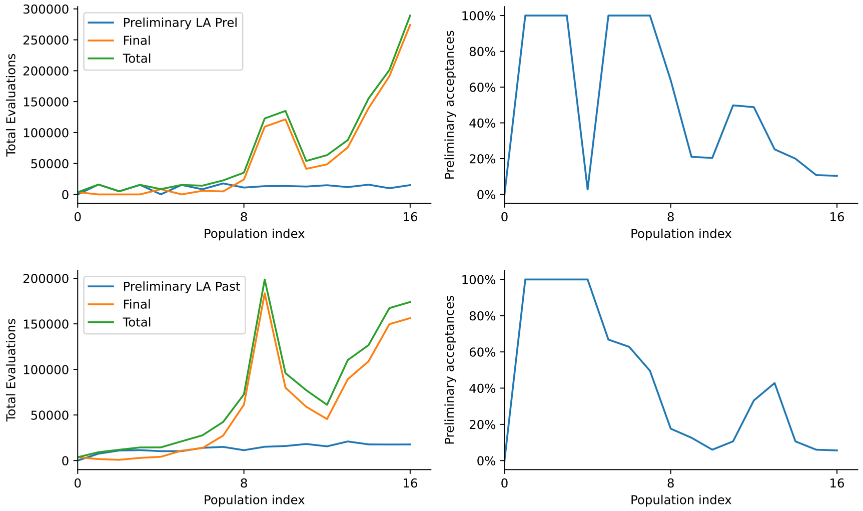

Figure S12: Total evaluations and fraction of accepted particles for (M2) based on the preliminary population in each generation in the LA Prel (top) and LA Past (bottom) runs of the same run as in Figure S11

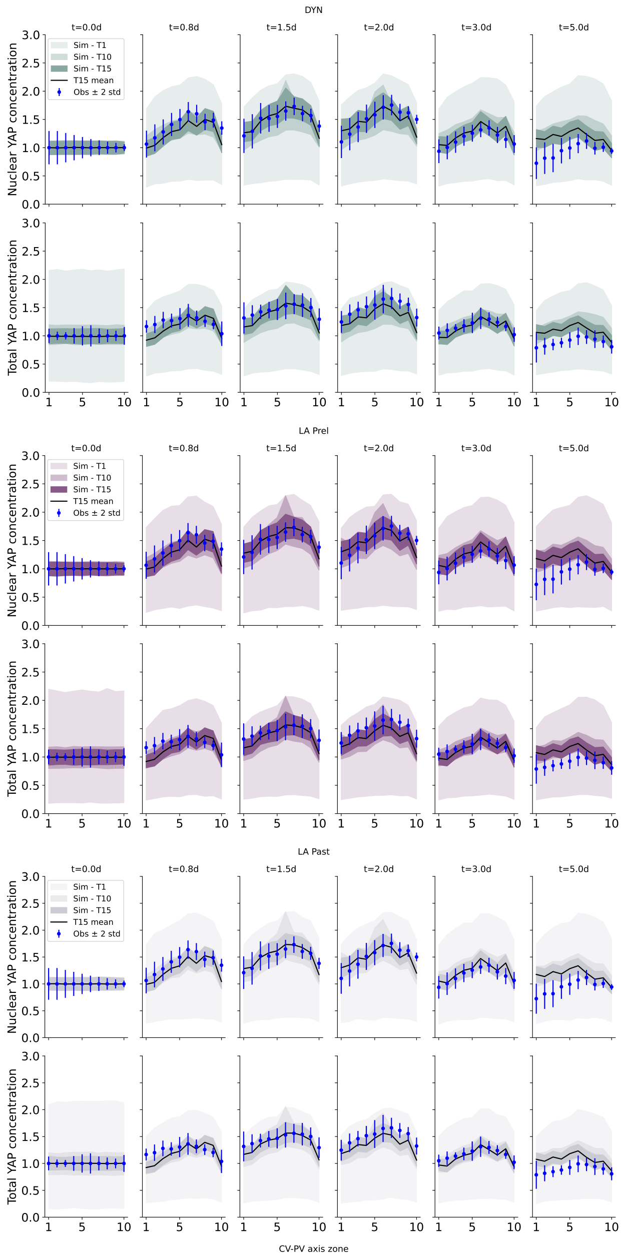

Figure S13: Comparison of best parameter fit for (M2) using DYN (top), LA Prel (middle), and LA Past (bottom) for a population size of N= 1000

Results – Run-Time

To save computational resources, we employed an early rejection strategy to reject particles based on a maximum run-time for individual simulations not 386 matching the data. However, this will decrease run-time heterogeneity. To assess the affect of that on the speed up of the look-ahead sampler, we fit the model using two different maximum run-time, 15, and 30 minutes. The result of the 30min maximum run-time can be seen in Figure 6. In addition, the 15min maximum run-time

is presented in figure S14. Result shows clearly that if heterogeneity increases, one would expect higher speed-up from both LA Prel and LA Past compared to DYN sampler.

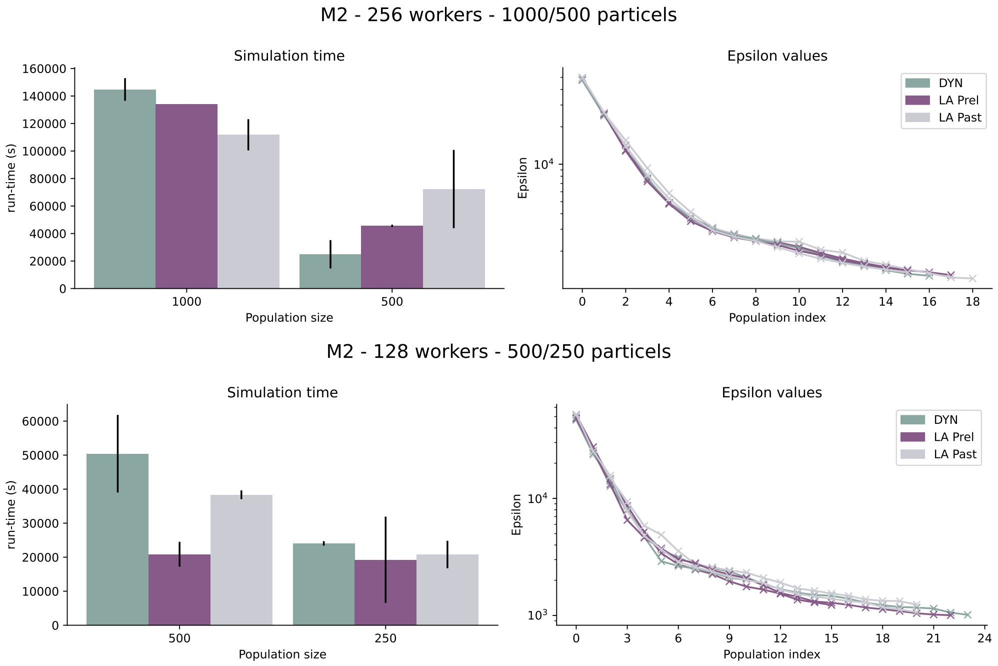

Figure S14: The run-time and posterior distributions for 2 different runs of the model M2 with population size 1000, 500, 250 on 128 and 256 workers. In this run, we use a maximum simulation time of 900 seconds, as opposed to 1900 seconds in Figure 5. We can see that decrease in heterogeneity level on simulation time drastically affected the effectiveness of the LA samplers.

Figure S15 shows the development of the epsilon threshold for the 8 different runs. In the more extreme cases, it also occurred that the LA scheduling took slightly longer than the corresponding DYN run, which was observed more in the less heterogeneous runs (e.g. when using 30min as maximum run-time).

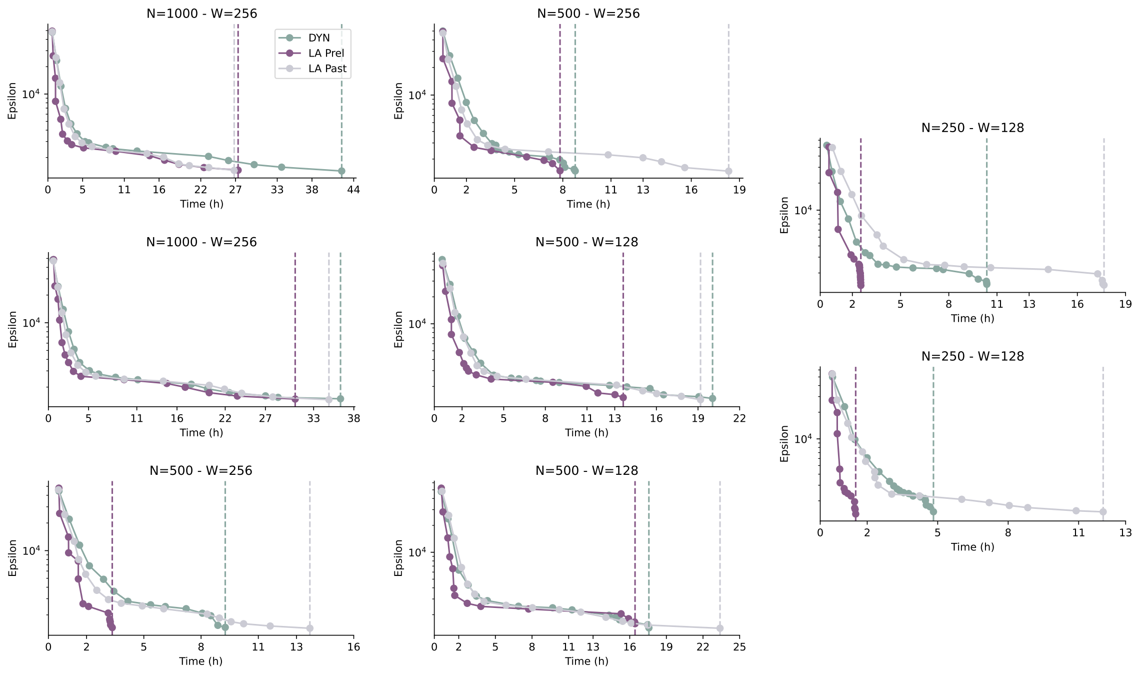

Figure S15: Development of the acceptance threshold over time for the different runs for model M2. These are all the 8 runs we performed.

On average, it seems that acceleration varies based on the population size and the number of workers to be used. Over the 8 times we have executed the inference of the liver regeneration model with an adaptive epsilon schedule, we observed a mean acceleration of 36%, with the median value being 31% for the LA Prel.

3.4.1 Simulation time variability

The simulation time for a model often relies on the parameter space, as exemplified by model M2. The forward simulation of this model is heavily influenced by the chosen set of parameters. For instance, parameters such as the inactivation rate of YAP (k4) or the activation rate of YAP (k5) both greatly affect the computation time, as demonstrated in Figure S16.

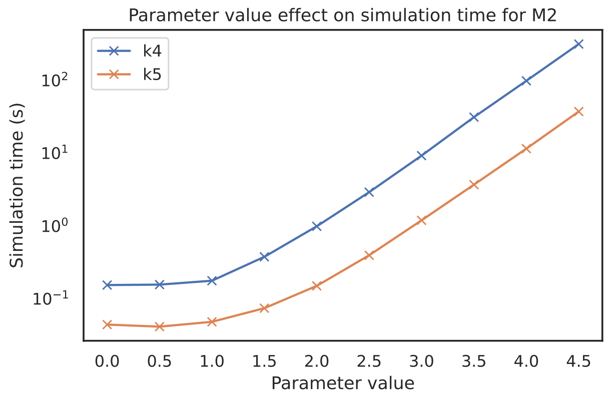

Figure S16: Simulating the liver regeneration model M2 using different values for the parameters k4 and k5 that range from 0 to 4.5 in log10 space

References

[1] J. Starruß, W. de Back, L. Brusch, and A. Deutsch. Morpheus: A user-friendly modeling environment for multiscale and multicellular systems biology. *Bioinf.*, 30(9):1331–1332, Jan. 2014.

[2] N. Jagiella. *Parameterization of lattice-based tumor models from data.* Ph.d. thesis, Université Pierre et Marie Curie, Paris, France, 2012.

[3] N. Jagiella, D. Rickert, F. J. Theis, and J. Hasenauer. Parallelization and high-performance computing enables automated statistical inference of multi-scale models. *Cell Syst.*, 4(2):194–206, 02 2017.

[4] K. Carver, X. Ming, and R. L. Juliano. Multicellular tumor spheroids as a model for assessing delivery of oligonucleotides in three dimensions. *Molecular Therapy – Nucleic Acids*, 3(e153), Mar. 2014.

[5] K. Kwapiszewska, A. Michalczuk, M. Rybka, R. Kwapiszewski, and Z. Brzózka Z. A microfluidic-based platform for tumour spheroid culture, monitoring and drug screening. *Lab Chip*, 14(12):2096–2104, June 2014.

[6] Kirstin Meyer, Hernan Morales-Navarrete, Sarah Seifert, Michaela Wilsch-Braeuninger, Uta Dahmen, Elly M Tanaka, Lutz Brusch, Yannis Kalaidzidis, and Marino Zerial. Bile canaliculi remodeling activates yap via the actin cytoskeleton during liver regeneration. *Mol. Syst. Biol.*, 16(2):e8985, 2020.
