# Supplementary material for: A wall-time minimizing parallelization strategy for approximate Bayesian computation
Source: PLoS One. 2024 Feb 22;19(2):e0294015. doi: 10.1371/journal.pone.0294015 (PMC10883530; doi:10.1371/journal.pone.0294015)
Supplement: Attachment — Submitted filename: LookAheadABC_Response.pdf [file pone.0294015.s002.pdf]

# Author's response to editor's and reviewers' comments

For the paper: "*A Wall-time Minimizing Parallelization Strategy for Approximate Bayesian Computation*".

We thank the editor and the reviewers for their comments. Based on their remarks, we have revised the manuscript and the Supplementary Material. We addressed all their comments and resolved the corresponding issues. Our point-by-point response is provided below.

The teal boxes contain the editor's and the reviewers' comments, while the yellow boxes contain the changes made in the manuscript. To provide some context, changes (teal) are sometimes accompanied by unchanged text (black).

## Response to Reviewer 1

### Summary

The article "A Wall-time Minimizing Parallelization Strategy for Approximate Bayesian Computation" proposed a Look-ahead scheduling technique to fully utilize the idle time that address issues the current static and dynamic scheduling have. Two kinds of LA scheme, LA pre and LA cur are proposed and compared through numerous numerical examples. t is clearly written and will be a useful contribution to the community. link to the software is provided. I was able to access the code through the link provided in the paper. A few minor comments for the manuscript that will be useful for the authors to discuss but does not require any further calculations for this paper, are as below.

We thank the reviewer for the feedback and the positive assessment of the quality.

As suggested, we made some modifications to the manuscript, as detailed in the point-to-point responses below.

### General comments

(1) In section 2.4.1, could the authors add more discussion about the bias introduced by LA Pre, such as, where does the bias come from, why particles with faster simulation times tend to introduce a practical bias?

We thank the reviewer for pointing out the lack of clarity. We have elaborated on the bias origin on Section 2.4.1 as follows:

*Changes in the manuscript:*

*Initial submission:*
However, $\hat{P}_{t-1}$ may introduce a practical bias (in a finite sample sense) towards particles with faster simulations times. This can in particular occur when computation time is highly parameter-dependent.

*Revised submission:*
However, $\hat{P}_{t-1}$ may introduce a practical bias (in a finite sample sense) towards particles with faster simulations times. This can in particular occur when computation time is highly parameter-dependent. Say, for example, that parameters from multiple regions in parameter space can explain the data similarly well, but that one region leads to substantially higher simulation times. Then, a sampling routine that does not wait for all started simulations to finish may under-represent or even miss out on regions in parameter space with high simulation times. The ABC-SMC routine may consequently have a low probability of generating importance samples from that region in subsequent generations, leading to a biased final posterior sample.

Further, we have extended Section 2.4.2 by the following remark:

*Changes in the manuscript:*

*Initial submission:*
The point is that each subpopulation on its own gives an asymptotically unbiased estimator, since the weights $\tilde{w}_t(\tilde{\theta}) = \pi(\tilde{\theta})/\tilde{g}_t(\tilde{\theta})$, $w_t(\theta) = \pi(\theta)/g_t(\theta)$ are exactly the Radon-Nikodym derivatives w.r.t. the respective proposal distributions. These estimates are then combined, which decreases the Monte Carlo error due to the larger sample size

*Revised submission:*
The point is that each subpopulation on its own gives an asymptotically unbiased estimator, since the weights $\tilde{w}_t(\tilde{\theta}) = \pi(\tilde{\theta})/\tilde{g}_t(\tilde{\theta})$, $w_t(\theta) = \pi(\theta)/g_t(\theta)$ are exactly the Radon-Nikodym derivatives w.r.t. the respective proposal distributions. Note that this theoretical unbiasedness holds regardless of whether $\tilde{g}_t$ is based on $\hat{P}_{t-1}$ or $P_{t-2}$, as long as $\tilde{g}_t(\theta) \gg \pi(\theta)$ As noted in the previous section, however a practical bias may occur due to finite sample size.
The subpopulation estimates are then combined, which decreases the Monte Carlo error due to the larger sample size.

(2) In Figure 3, the authors illustrate the bias caused by LA pre scheduling method. However, it's hard for me to tell the information from the right hand-side plot. I was wondering if a more detailed explanation for the problematic realization (the purple "vertical" lines), or updated plots with extended y-axis can be posted?

We thank the reviewer for this suggestion. We think that extending the y-axis will indeed improve the understanding of the curves as probability densities. We have consequently updated Figure 3 as follows:

*Changes in the manuscript:*

*Initial submission:*

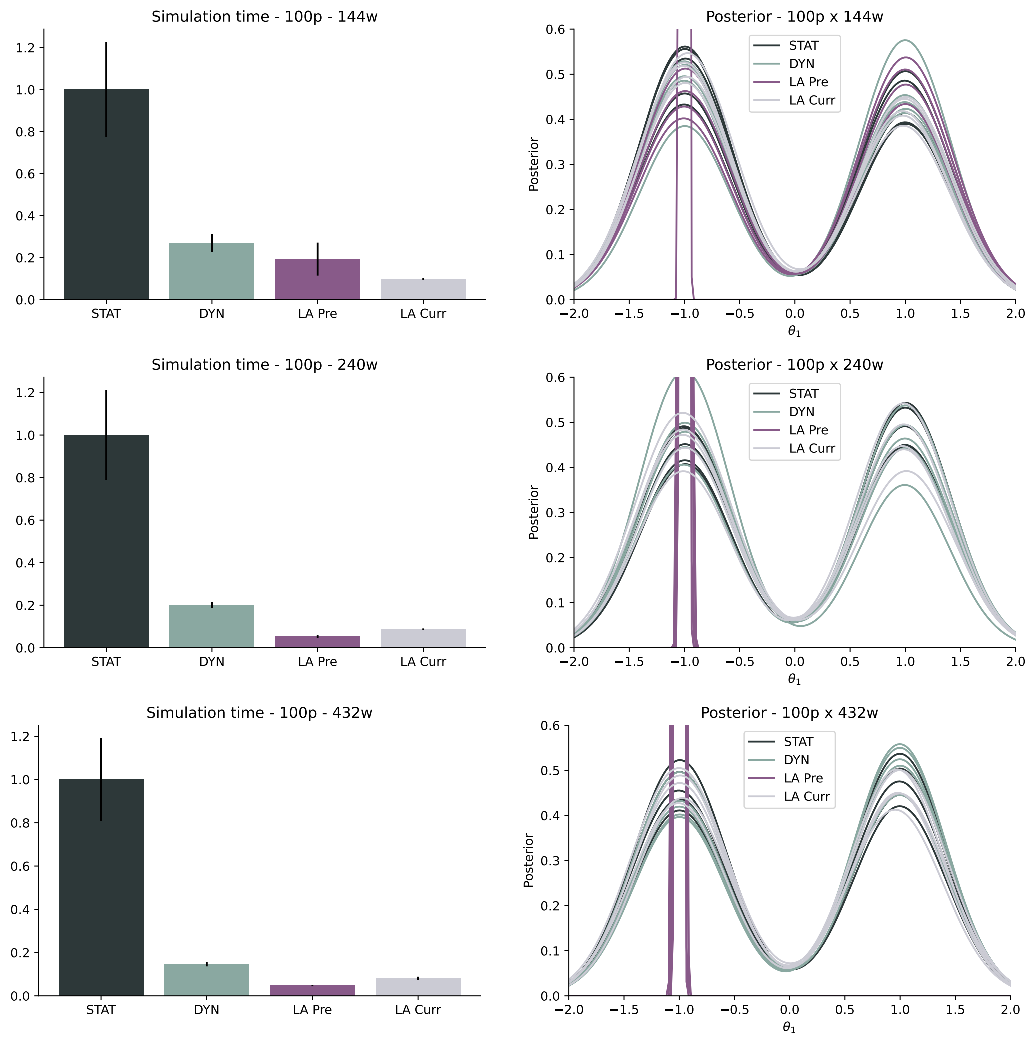


*Revised submission:*

(3) In section 3.5, what's the reason behind the slower performance as the ratio between number of population size and number of workers increase?

We assume that this comment refers to Figure 5, showing the speed-up of LA over DYN for different population sizes $N$ and numbers of workers $W$. The figure shows that with increasing $W$ (relatively) the speed-up generally increases, while with increasing $N$ (relatively) the speed-up generally decreases. We have added the following explanation to Section 3.5:

*Changes in the manuscript:*

*Initial submission:* Only when the population size was large compared to the number of workers, the speed-up was comparably small.

*Revised submission:* Only when the population size was large compared to the number of workers, the speed-up was comparably small. Generally, the increase in speed-up with increasing ratio $W/N$ is as expected, as for large $W$ the idle time between generations occurring for DYN and LA constitutes a more pronounced factor in the overall run-time.

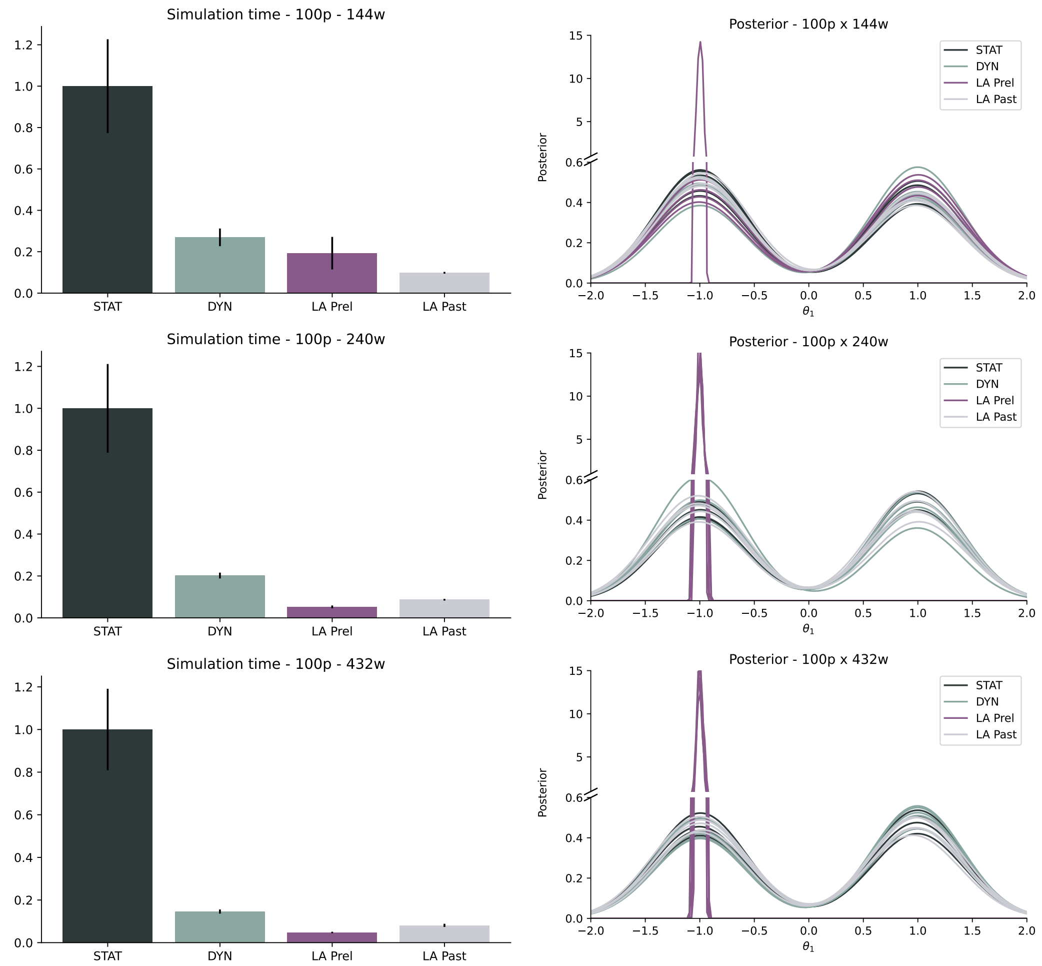


(4) How do you determine which method (LA pre and LA cur) is the better choice given a problem?

We thank the reviewer for this comment. We have added our recommendation to the Discussion:

*Changes in the manuscript:*

*Initial submission:* We observed that look-ahead sampling using preliminary results (LA Pre) provided a performance speed-up over re-using the previous generation (LA Cur), however at the cost of practical bias.

*Revised submission:*
We observed that look-ahead sampling using preliminary results (LA Prel) provided a performance speed-up over re-using the previous generation (LA Past), however at the cost of practical bias. Thus, LA Past constitutes the safe choice. Only if the possibility of critical parameter-dependent simulation times can be excluded, would we presently recommend LA prel.

## Response to Reviewer 2

### Summary

In this study, E. Alamoudi and his colleagues develop a novel parallelization strategy implemented in an high-performance computing (HPC) infrastructure. This strategy aims at improving the speed, mainly by reducing wall-time, of Approximate Bayesian Computation (ABC) sequential Monte Carlo algorithm. The authors show that their new strategy is unbiased and converges to the expected value of Monte Carlo sampling. They also compare their strategy (look-ahead scheduling) with existing parallelization strategies, assessing relative performance at four different test problems. Given its flexibility, ABC is a popular approach that can be applied to very complex models. As such, any potential speed-up strategy is useful and could prove to be valuable for many researchers. Thus, it is my recommendation that this manuscript should be accepted. Nevertheless, I have a few comments and suggestions. Please note that, although I have worked with ABC before, I am not a computational scientist and thus all my comments will be aimed at improving the clarity of the manuscript for a more general audience. I hope my comments are useful and contribute to make this submission more valuable for PlosOne wide readership.

We thank the reviewer for the assessment and for pointing out the practical relevance.

### Major comments

(1) I think the authors limit the scope of the manuscript by starting the introduction with systems biology. Although "systems biology" is an umbrella term that encompasses several fields and research questions, this parallelization strategy could be useful in other fields such as population genomics or human health, for instance. I think a more general start is warranted, possibly by just mentioning the variety of applications of ABC, including systems biology.

We thank the reviewer for this comment. Indeed, while our work is mainly in systems biology, ABC finds application far beyond that. To better reflect this wide range of potential applications, we have modified the introduction as follows:

*Changes in the manuscript:*

*Initial submission:* The ultimate goal of systems biology is a holistic understanding of biological systems [1, 2]. Mechanistic models are important tools towards this aim, allowing to describe and understand underlying mechanisms [3]. Usually, such models have unknown parameters that need to be estimated by comparing model outputs to observed data [4].

*Revised submission:* Mechanistic models are important tools in systems biology and many other research fields to describe and understand mechanisms underlying systemic behavior [1, 3]. Usually, such models have unknown parameters that need to be estimated by comparing model outputs to observed data [4].

(2) "While asymptotically exact, a known disadvantage of ABC is its reliance on repeated simulation, often hundred thousands to millions of times" - while this is technically correct, further ahead the authors clearly state the 3 steps of classical ABC. Thus, I think this could be rephrased to highlight that a disadvantage of ABC is the computationally heavy and time consuming simulation of data points (or datasets), which is usually the most time consuming step of ABC.

We thank the reviewer for this comment. We believe that further expansion would improve the reader's understanding of this aspect of ABC:

*Changes in the manuscript:*

*Initial submission:* While asymptotically exact, a known disadvantage of ABC is its reliance on repeated simulation, often hundred thousands to millions of time

*Revised submission:*
While asymptotically exact, a known disadvantage of ABC is its computational complexity. The reason for this is that it requires often simulations of hundred thousands to millions of artificial datasets

(3) I believe there is an unfinished sentence in the "ABC-SMC" section of the Methods. The sentence that starts with "Methods that adapt to the problem structure" ends with "have shown superior". I think a word is missing after "superior".

We thank the reviewer for this observation. We corrected this mistake in the revised version.

*Changes in the manuscript:*

*Initial submission:* Methods that adapt to the problem structure, e.g. basing $\Sigma_{t-1}$ on the previous generation's weighted sample covariance matrix and potentially localizing around $\theta_i$, have shown superior [5–7]. .

*Revised submission:* Methods that adapt to the problem structure, e.g. basing $\Sigma_{t-1}$ on the previous generation's weighted sample covariance matrix and potentially localizing around $\theta_i$, have shown superior performance [5–7].

(4) Although Figure 1 is useful to understand the comparison between the different parallelization strategies, I found it somewhat confusing. Particularly, it was confusing to see shades of gray in the Figure key but not in the Figure itself. I think the authors could remove the shading from the Figure key and include that information only in the Figure legend itself. Possibly as: "The shading associated with each color indicates whether a sample satisfies the acceptance criterion and is included in the final population (dark shading), satisfies the acceptance criterion but is discarded because enough earlier-started accepted samples exist (intermediate shading, for DYN+LA), or does not satisfy the acceptance criterion and is rejected (light shading)." Additionally, it is not clear what the blank areas in-between shaded areas represent for each worker? Idle times or post-process?

We thank the reviewer for this observation. We have included the suggested clarification of the shading in the caption. When testing versions of the figure with and without the shades of grey in the legend, the version without was found to take longer to understand, thus we suggest to keep it, in addition to the clarified legend. The modified caption, including clarifications on shading and white spaces, is below:

*Changes in the Supplementary Material:*

*Initial submission:*

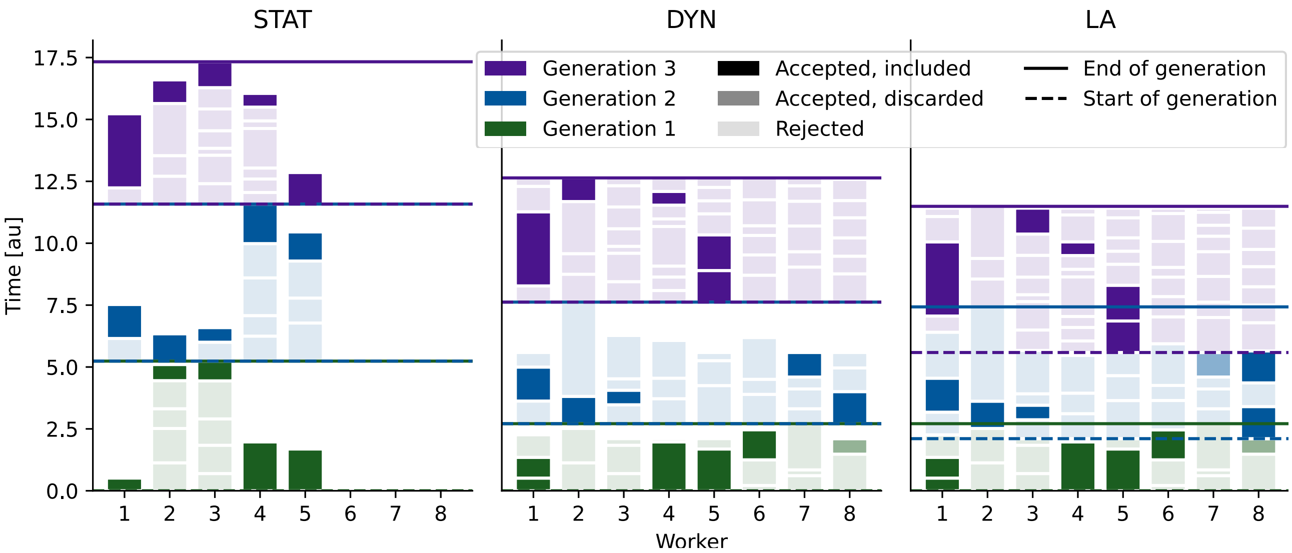


Figure 1: Illustration of core usage over run-time for static (STA), dynamic (DYN) and look-ahead (LA) scheduling for a population size $N = 5$ on $W = 8$ workers, over 3 generations (colors). The shading indicates whether a sample satisfies the acceptance criterion and is included in the final population (dark), satisfies the acceptance criterion but is discarded because enough earlier-started accepted samples exist (medium, for DYN+LA), or does not satisfy the acceptance criterion and is rejected (light). Solid lines indicate the end of a generation, dashed lines indicate the (preliminary) beginning of a generation (different from solid lines only for LA).

*Revised submission:*

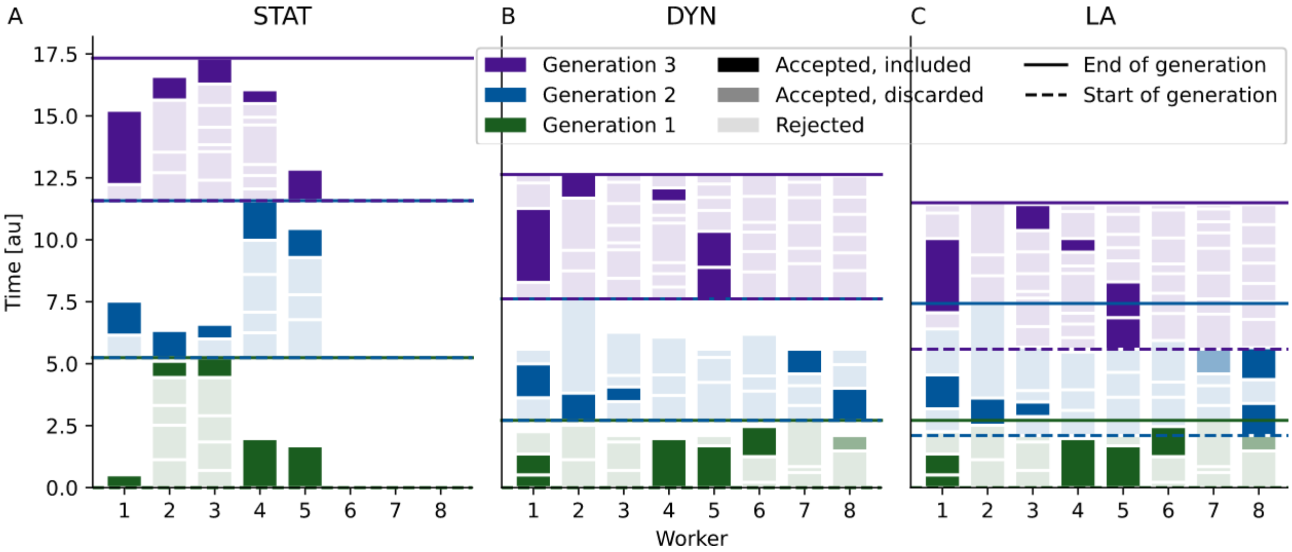


Figure 2: Illustration of core usage over run-time for static (STA), dynamic (DYN) and look-ahead (LA) scheduling for a population size $N = 5$ on $W = 8$ workers, over 3 generations (colors). The shading associated with each color indicates whether a sample satisfies the acceptance criterion and is included in the final population (dark shading), satisfies the acceptance criterion but is discarded because enough earlier-started accepted samples exist (intermediate shading, for DYN+LA), or does not satisfy the acceptance criterion and is rejected (light shading). Solid lines indicate the end of a generation, dashed lines indicate the (preliminary) beginning of a generation (different from solid lines only for LA). Delimiting white spaces between boxes indicate negligible post-processing times between simulations.

(5) Throughout the text the authors refer to Figure 1A, 1B and 1C but I did not see any A, B or C indicated in Figure 1.

We thank the reviewer for this comment. The updated figure with the sub-figure names is introduced in the answer of comment 4.

(6) When explaining static scheduling (STAT), the authors mention that the "tasks are queued if $N \geq W$". Do tasks really get queued if $N = W$? It was my understanding that if N workers are available then all tasks will start and no task will be queued.

We thank the reviewer for pointing out to the unintentional mistake. True, the statement should be only $N > W$.

*Changes in the manuscript:*

*Initial submission:* The tasks are queued if $N \geq W$..

*Revised submission:* The tasks are queued if $N > W$.

(7) "Therefore, DYN waits for all workers to finish, and out of then $\tilde{N} \geq N$ accepted particles, only the N that started earliest are finally accepted". Is this correct? This sentence is confusing, because if there is a bias towards accepting particles that end earlier, how would waiting for all workers to finish and then selecting the N that started earlier solve that bias?

We thank the reviewer for this feedback. By waiting for all particles to finish, we are only concerned about the starting time of each particle, not its computational heaviness. By doing so, we eliminate the bias toward faster simulation, which would occur if we simply accept the

first $N$ particles that have finished. We have modified the statement as follows:

*Changes in the manuscript:*

*Initial submission:* Therefore, DYN waits for all workers to finish, and out of then $\tilde{N} \geq N$ accepted particles, only the $N$ that started earliest are finally accepted [8]. This ensures that the acceptance probability of a particle is in accordance with the target distribution, independent of later events and thus its run-time.

*Revised submission:*
Therefore, DYN waits for all workers to finish, and out of then $\tilde{N} \geq N$ accepted particles, only the $N$ that started earliest are finally accepted, not the ones that finished earliest. This has the effect that simulation time plays no longer a role in the acceptance decision [8]. This ensures that the acceptance probability of a particle is in accordance with the target distribution, independent of later events and thus its run-time.

(8) This might just be a personal preference but I don't understand the rationale behind the naming convention chosen for "LA Cur" and "LA Pre". Although I understand that "LA Pre" refers to the use of a Preliminary sample, "LA Cur" makes me think of "Current" which is less than intuitive given that it is using an earlier generation Pt-2. Maybe this will be clearer for other readers but, for me, it would make more sense to call these two approaches "LA Pre" for preliminary and "LA Pge" for the past generation.

We thank the reviewer for this feedback. We totally agree with the suggestion. A better naming scheme will reflect better understanding by the reader. We revised the text, with the following short notes used consistently throughout the text:

"LA Cur" → "LA Past" and "LA Pre" → "LA Prel"

*Changes in the manuscript:*

*Initial submission:* "LA Cur" "LA Pre"

*Revised submission:* "LA Cur" → "LA Past" and "LA Pre" → "LA Prel"

(9) In the subsection "Test problems", I think a few more general details about the problems considered would make the results more understandable to a wider audience. I suggest adding a table with the parameters for each problem to the supplementary information. The authors should also mention that a detailed explanation of the test problems can be found in the supplementary information. Additionally, I think the authors should expand their explanation of T1 since this problem is important to understand the preliminary bias associated with "LA Pre". Particularly, I think that they should more clearly state that the aim of the problem is to infer a posterior distribution with two modes (i.e., a bimodal distribution), which is a tricky problem. Furthermore, it could be made clearer that the goal of setting an artificially longer run-time was to specifically test the preliminary bias caused by only accepting simulations from one mode when constructing gt based on Pt-1.

We thank the reviewer for this feedback. We added additional details regarding the test problems as follows:

*Changes in the manuscript:*

*Initial submission:* Problem T1 is a bimodal model $y \approx \theta^2$, in which simulations from one mode have an artificially longer run-time, reflecting parameter dependency of run-times. Problem T2 is an ordinary differential equation (ODE) model with 2 parameters describing a conversion reaction $x_1 \leftrightarrow x_2$, with observables obscured by random multiplicative noise. To analyze sampler behavior under simulation run-time heterogeneity, we added random log-normally distributed delay times $t_{\text{sleep}}$ of various variances on top of the ODE simulations. For this model, run-times are fast, permitting repeated analyses to check correctness of the method, quantify stochastic effects and assess average behavior. Problem M1 describes the growth of a tumor spheroid using a hybrid discrete-continuous approach, modeling single cells as stochastically interacting agents and extracellular substances via PDEs [9]. Problem M2 describes the metabolic status of mechano-sensing during liver generation, describing the reaction network dynamics by a set of ODEs [10]. .

*Revised submission:*
Problem T1 is a bimodal model $y \approx \theta^2$, in which simulations from one mode have an artificially longer run-time. Specifically, if $\theta > 0$, a log-normally distributed simulation time of $\tau \sim \log \mathcal{N}(1, 2, 4)$ seconds was simulated. The goal of setting an artificially longer run-time was to specifically test the preliminary bias caused by only accepting simulations from one mode when constructing the preliminary proposal.
Problem T2 is an ordinary differential equation (ODE) model with 2 parameters describing a conversion reaction $x_1 \leftrightarrow x_2$, with observables obscured by random multiplicative noise. To analyze sampler behavior under simulation run-time heterogeneity, we added random log-normally distributed delay times $t_{\text{sleep}}$ of various variances on top of the ODE simulations. For this model, run-times are fast, permitting repeated analyses to check correctness of the method, quantify stochastic effects and assess average behavior.
Problem M1 describes the growth of a tumor spheroid using a hybrid discrete-continuous approach, modeling single cells as stochastically interacting agents and extracellular substances [9]. The model combines a system of PDEs to describe the extracellular matrix, with a cellular Potts model (CPM) to describe cell configurations and mechanisms like cell division and cell death. The model has seven estimated parameters and outputs three observables, the growth curve, extra-cellular matrix and proliferation profiles.
Problem M2 describes the metabolic status of mechano-sensing during liver generation, describing the reaction network dynamics by a set of ODEs [10]. This model has 14 parameters and two observables, the nuclear YAP and total YAP intensities. These observables were quantified from image tiles covering an entire liver lobule with portal and central veins.
Further details about the test problems can be found in the Supplementary Information, Section 3.

In addition, we added the following tables that describe the parameter list to the Supplementary Information as follows:

*Changes in the manuscript:*

*Initial submission:*

*Revised submission:*
Parameter table for model (T1)

*Revised submission:*
Parameter table for model (T2)

Table 1: List of fitted parameters of model T1. All parameters have been transformed to a logarithmic scale with base 10.

| Parameter | Description | Prior |
|---|---|---|
| $\log_{10}(\theta)$ | A squared parameter | U(-2,-4) |

Table 2: List of fitted parameters of model T2. All parameters have been transformed to a logarithmic scale with base 10.

| Parameter | Description | Prior |
|---|---|---|
| $\log_{10}(\theta_1)$ | Conversion rate of $x_1$ | $U(0,1)^2$ |
| $\log_{10}(\theta_2)$ | Conversion rate of $x_2$ | $U(0,1)^2$ |

*Revised submission:*
Parameter table for model (M1)

Table 3: List of fitted parameters of model M1. All parameters have been transformed to a logarithmic scale with base 10.

| Parameter | Description | Prior |
|---|---|---|
| $\log_{10}(k_{\text{max}}^{\text{div}})$ | Division rate | U(-3,-1) |
| $\log_{10}(L_{\text{div}})$ | Division depth | U(-5,0) |
| $\log_{10}(L_{\text{init}})$ | Initial spheroid radius | U(1,3) |
| $\log_{10}(q_{\text{init}})$ | Initial quiescent cell fraction | U(0,1.2) |
| $\log_{10}(k_{\text{pro}}^{e})$ | ECM production rate | U(-5,0)) |
| $\log_{10}(k_{\text{deg}}^{e})$ | ECM degradation rate | U(-5,0) ) |
| $\log_{10}(e_{\text{div}})$ | ECM division threshold | U(-5,0)) |

*Revised submission:*
Parameter table for model (M2)

(10) In the section titled ”Sampling from unbiased proposal solves bias” the authors correctly state that ”LA Pre may fail in some situations, which also demonstrates that ABC-SMC algorithms are sensitive to potential bias in the proposal distribution”. Given sufficient number of generations, is it possible to use ”LA Cur” in the first generations, as a sort of ”burn-in”, and then switch to ”LA Pre” when the probability of a bias towards short-running simulations is lessened?

We thank the reviewer for this feedback. Indeed, such an approach appears reasonable, albeit demanding further empirical justification. We have extended the discussion as follows:

*Changes in the manuscript:*

*Initial submission:* Were it possible to construct an unbiased proposal using those preliminary results, e.g. via reweighting or imbalance detection, we could thus increase the speed-up with robust performance.

*Revised submission:* Were it possible to construct an unbiased proposal using those preliminary results, e.g. via reweighting or imbalance detection, we could thus increase the speed-up with robust performance. Alternatively, LA Past and LA Prel could also be combined, e.g. switching to LA Prel after a “burn-in”, when the probability of a bias toward short-running simulations is lessened.

Table 4: List of fitted parameters of model M2. All parameters have been transformed to a logarithmic scale with base 10.

| Parameter | Description | Prior |
|---|---|---|
| $\log_{10}$(k1) | Max. flux of SENSOR activation | U(1,3) |
| $\log_{10}$(k2) | Max. flux of SENSOR inactivation | U(0,2) |
| $\log_{10}$(k3) | Factor of YAP synthesis rate | U(-0.76,1.23) |
| $\log_{10}$(k4) | YAP inactivation rate | U(-1.95,0.04) |
| $\log_{10}$(k5) | YAP activation rate | U(1,4) |
| $\log_{10}$(k6) | Inact. YAP binding rate to SF | U(-1.74,0.25) |
| $\log_{10}$(k7) | Inact. YAP unbin. rate from SF | U(1,4) |
| $\log_{10}$(k8) | YAP export rate from nucleus | U(1,4) |
| $\log_{10}$(k9) | YAP import rate into nucleus | U(-1.76,0.23) |
| $\log_{10}$(k10) | YAP degradation rate | U(-0.95,1.04) |
| $\log_{10}$(k11) | Inact. YAP degradation rate | U(0.47,2.47) |
| $\log_{10}$(k12) | M-M const. of SENSOR activation | U(-4,-2) |
| $\log_{10}$(k13) | M-M const. of SENSOR inactivation | U(-1.6,0.40) |
| $\log_{10}$(k14) | Intensity normalization total | U(-1,1) |

(11) In section "3.6 Scales to realistic application problems", is it possible that the "mean slow-down of 39% (median 43%) over DYN" observed for "LA Cur" is a result of a slower refinement of the preliminary proposal distribution? In other words, for a sufficiently complex model, basing the proposal distribution on Pt2 implies that the search space for each generation is more vast than it would be if proposal distribution was based on Pt1. Could this explain the observed slow-down?

We thank the reviewer for this observation. Indeed, this is also what we hypothesize to be a likely cause, although the substantial variability of run-times across replicates does not allow to make such a statement with clarity.

*Changes in the manuscript:*

*Initial submission:* Were it possible to construct an unbiased proposal using those preliminary results, e.g. via reweighting or imbalance detection, we could thus increase the speed-up with robust performance.

*Revised submission:* Were it possible to construct an unbiased proposal using those preliminary results, e.g. via reweighting or imbalance detection, we could thus increase the speed-up with robust performance. Alternatively, LA Past and LA Prel could also be combined, e.g. switching to LA Prel after a "burn-in", when the probability of a bias toward short-running simulations is lessened. However, such approaches would require further careful evaluation.

# References


[1] H. Kitano. Systems biology: A brief overview. Science, 295(5560):1662–1664, 03 2002.

[2] J. R. Karr, J. C. Sanghvi, D. N. Macklin, M. V. Gutschow, J. M. Jacobs, B. Bolival Jr, N. Assad-Garcia, J. I. Glass, and M. W. Covert. A whole-cell computational model predicts phenotype from genotype. Cell, 150(2):389–401, July 2012.

[3] Neil A Gershenfeld and Neil Gershenfeld. The nature of mathematical modeling. Cambridge university press, 1999.

[4] A. Tarantola. Inverse Problem Theory and Methods for Model Parameter Estimation. SIAM, 2005.

[5] Mark A Beaumont, Jean-Marie Cornuet, Jean-Michel Marin, and Christian P Robert. Adaptive approximate bayesian computation. Biometrika, 96(4):983–990, 2009.

[6] S. Filippi, C. P. Barnes, J. Cornebise, and M. P. Stumpf. On optimality of kernels for approximate Bayesian computation using sequential Monte Carlo. Stat. Appl. Genet. Mol., 12(1):87–107, 2013.

[7] E. Klinger and J. Hasenauer. A scheme for adaptive selection of population sizes in Approximate Bayesian Computation - Sequential Monte Carlo. In J. Feret and H. Koeppl, editors, Computational Methods in Systems Biology. CMSB 2017, volume 10545 of Lecture Notes in Computer Science. Springer, Cham, 2017.

[8] Emmanuel Klinger, Dennis Rickert, and Jan Hasenauer. pyABC: distributed, likelihood-free inference. Bioinf., 34(20):3591–3593, 10 2018.

[9] N. Jagiella, D. Rickert, F. J. Theis, and J. Hasenauer. Parallelization and high-performance computing enables automated statistical inference of multi-scale models. Cell Syst., 4(2):194–206, 02 2017.

[10] Kirstin Meyer, Hernan Morales-Navarrete, Sarah Seifert, Michaela Wilsch-Braeuninger, Uta Dahmen, Elly M Tanaka, Lutz Brusch, Yannis Kalaidzidis, and Marino Zerial. Bile canaliculi remodeling activates yap via the actin cytoskeleton during liver regeneration. Mol. Syst. Biol., 16(2):e8985, 2020.
